# Photoacoustic imaging for investigating tumor hypoxia: a strategic assessment

**DOI:** 10.7150/thno.84253

**Published:** 2023-05-29

**Authors:** Deyana Nasri, Rayyan Manwar, Ajeet Kaushik, Ekrem Emrah Er, Kamran Avanaki

**Affiliations:** 1 Richard and Loan Hill Department of Bioengineering, University of Illinois at Chicago, IL, USA.; 2 Department of Natural Sciences, Florida Polytechnic University, Lakeland, FL, USA.; 3 Department of Physiology and Biophysics, College of Medicine, University of Illinois at Chicago, IL, USA.; 4 Department of Dermatology, University of Illinois at Chicago, Chicago, IL, USA.

**Keywords:** Photoacoustic imaging, tumor hypoxia, angiogenesis, contrast agents, label-free imaging

## Abstract

Hypoxia causes the expression of signaling molecules which regulate cell division, lead to angiogenesis, and further, in the tumor microenvironment, promote resistance to chemotherapy and radiotherapy, and induce metastasis. Photoacoustic imaging (PAI) takes advantage of unique absorption characteristics of chromophores in tissues and provides the opportunity to construct images with a high degree of spatial and temporal resolution. In this review, we discuss the physiologic characteristics of tumor hypoxia, and current applications of PAI using endogenous (label free imaging) and exogenous (organic and inorganic) contrast agents. Features of various methods in terms of their efficacy for determining physiologic and proteomic phenomena are analyzed. This review demonstrates that PAI has the potential to understand tumor growth and metastasis development through measurement of regulatory molecule concentrations, oxygen gradients, and vascular distribution.

## 1. Introduction

**Figure 1:**
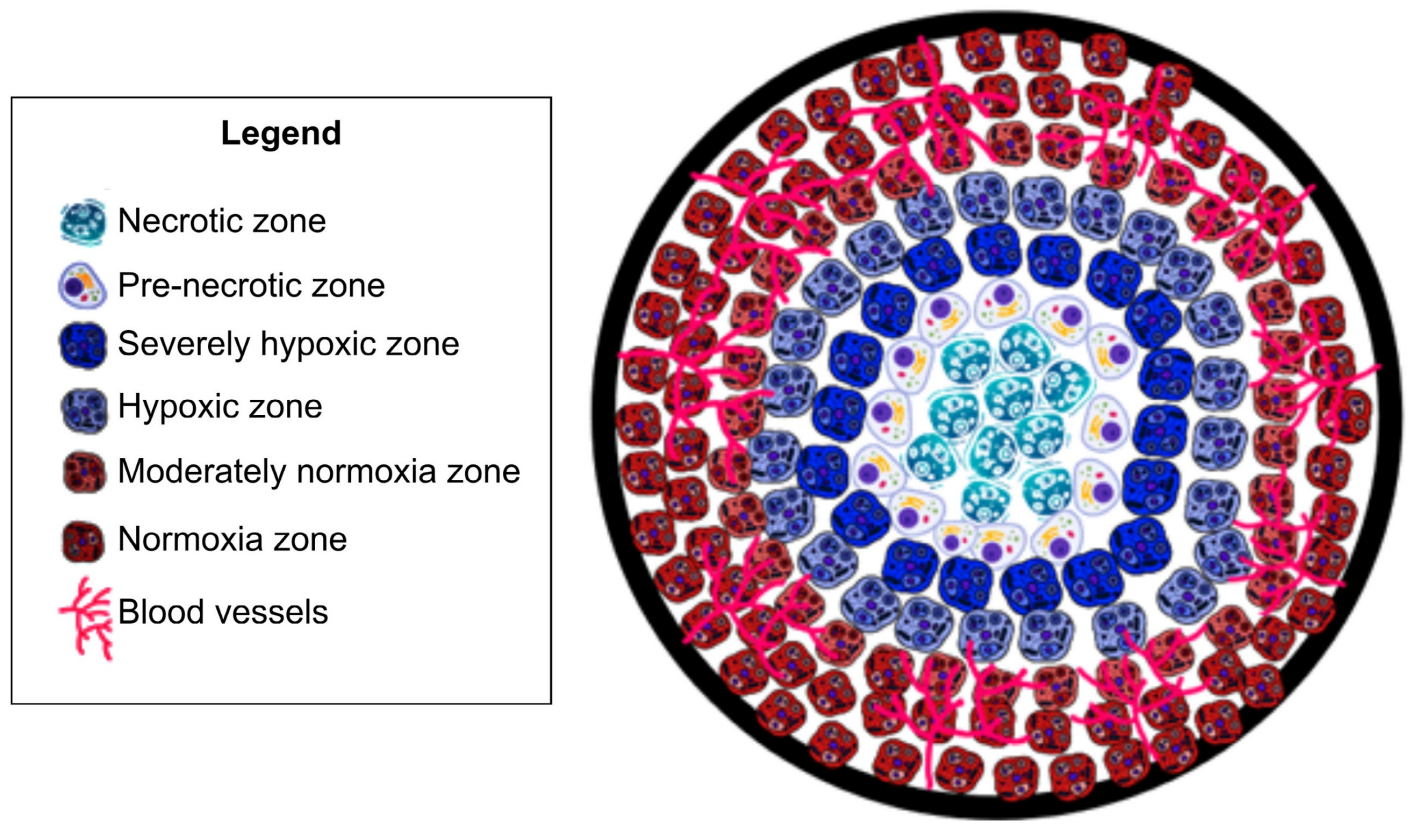
**Oxygen and vasculature distribution in tumor.** Schematic model of oxygen distribution in a typical solid tumor. As pictured, vasculature is concentrated in the periphery of tumor. As a result, the center/”core” of tumor is necrotic due to a lack of vasculature leading to insufficient oxygenation to keep cells viable. Moving out to periphery of the tumor, oxygenation levels increase. Leading to an increased level of oxygenation. As a result, there are an increased number of cells. This is due to the sufficient oxygenation promoting cell proliferation. This allows the tumor to grow and further secrete pro-angiogenic factors. Leading to more blood vessel formation, ultimately leading to metastasis.

Tumor hypoxia is a hallmark of the tumor microenvironment and is defined as a lack of oxygenation to the tumor tissue, due to insufficient blood supply 1. As tumors grow, excessive extracellular matrix produced by the tumor cells and the surrounding stromal fibroblasts physically compresses the tumor and normal vasculature, preventing proper perfusion and thereby inducing hypoxia 2. Hypoxia triggers release of angiogenic factors which are not evenly distributed throughout the tumor 3, 4. This uneven distribution leads to highly vascularized areas interspersed with areas of hypoxia 1, 3, 5. Due to this disordered vascularization, oxygenation is not consistent throughout the tumor 1, 3, which therefore displays significant heterogeneity of oxygen utilization 6. Abrupt and brief (of the order of minutes) exposure to hypoxia due to structural and functional abnormalities in the tumor microvasculature is considered 'acute' hypoxia 3, leading to tumor cell survival by activation of autophagic, apoptotic, and metabolic adaptation pathways and a decrease of oxidative metabolism 3. As hypoxic conditions increase to 'chronic' or 'diffusion limited' due to hyperproliferation of tumor cells, hypoxia further increases towards the tumor core 3, which surpasses the capacity of the vascular supply; leading to tumor cell exposure to hypoxic conditions for long periods 3 and ultimately leading to cell death and tumor necrosis (See Fig. 1). This is particularly important because acute and chronic hypoxia have different effects on tumor biology 3. Studies have shown that the outer components of a growing tumor are more oxygenated as compared to the core (center of tumor) due to an increased vascular density 1, 3, 5. Understanding the role of vascularization in tumor growth is important as dysregulation profoundly impacts tumor growth and ultimately cancer metastases 1, 3, 5. Dysregulated tumor angiogenesis and lack of perfusion further perpetuates hypoxia, and this dramatically alters the cancer cells' aggressive phenotypes. For example, hypoxia negatively impacts immune infiltration and composition allowing for immune evasion of tumor cells 7. Furthermore, in cancers of epithelial origin, tumor cells that experience hypoxia *in vitro* and *in vivo* upregulate hypoxia inducible transcription factors (HIF-1/2) and thereby undergo epithelial to mesenchymal transformation (EMT). EMT bestows tumor cells with more invasive and migratory phenotypes for enhanced metastases 3. Additionally, exposure of tumor cells to hypoxia in the primary tumor microenvironment activates survival signaling in tumor cells and these cells have a superior ability to remain dormant and alive even years after dissemination, akin to a ticking metastatic time-bomb 8. Thus, the impact of hypoxia on tumor progression results in a multitude of negative prognostic outcomes 9. In particular, epidemiological studies have shown cancer survival rates to decrease in patients with a higher degree of tumor hypoxia 9. Furthermore, cancer patients with higher levels of tumor hypoxia have shown greater resistance to chemotherapy and radiotherapy 10-12. Despite the plethora of research studies highlighting tumor hypoxia's impact on cancer prognosis; there is no comprehensive clinical method to assess tumor hypoxia 9.

**Figure 2:**
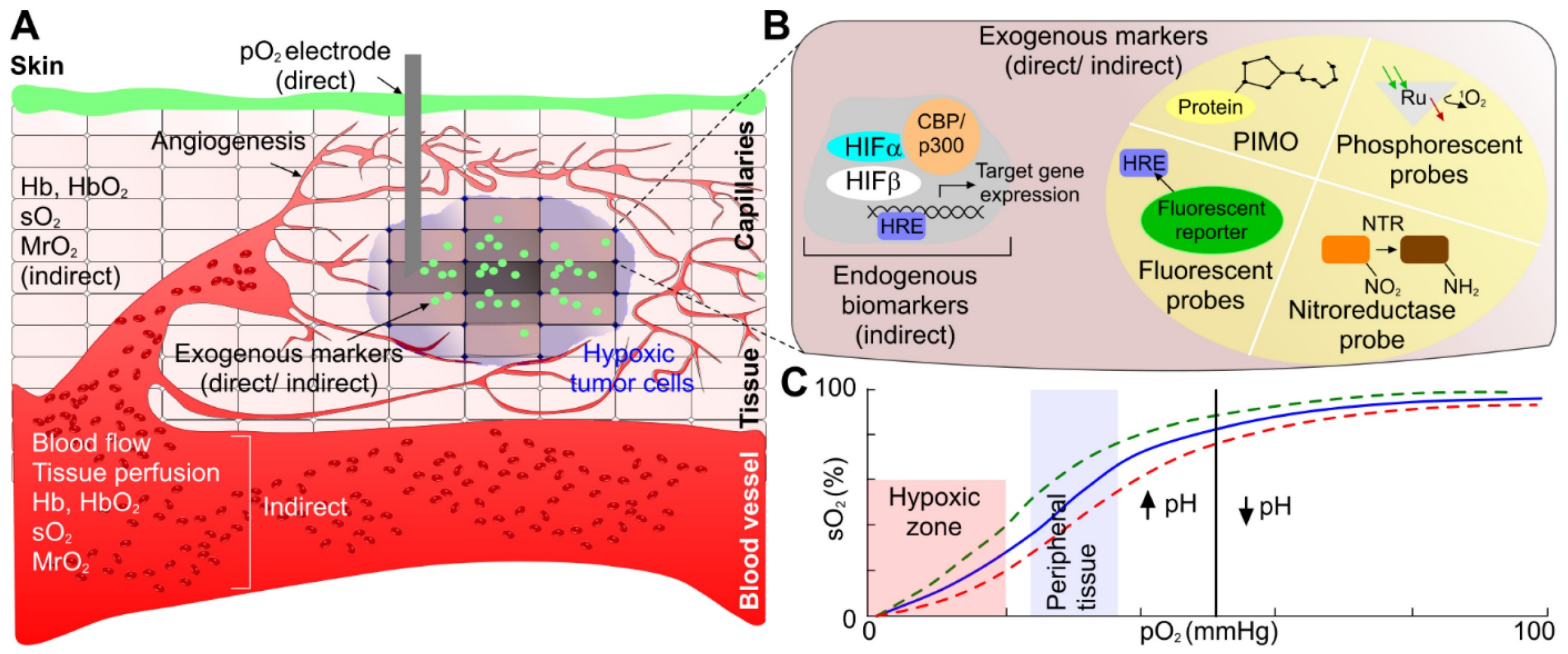
**Tumor hypoxia assessment mechanism. (a)** Proteomic/genomic transformation of endogenous biomarkers (i.e. HIF and other proteins) and their upregulation determine the hypoxic tumor progression path, physiological hemodynamic/metabolic alteration characterization using direct methods based on pO_2_ electrode and indirect method based on blood flow, perfusion, sO_2_, pO_2_, and MrO_2_ originating from endogenous chromophores (i.e., oxy- and deoxygenated hemoglobin concentration and ratio), and exogenous contrast agent activation and uptake. **(b)** An expanded view of one of the hypoxic tumor cells demonstrating both endogenous biomarkers (immunolabeling of HIF-1 or HIF-2 protein or immunolabeling of downstream transcriptional targets of HIFs proteins), and exogenous contrast agent-based tumor hypoxia assessment mechanism (immunolabeling of hypoxia probes - PIMO, Oxygen quenches phosphorescent probes 2-photon light, DNA construct is transcriptionally regulated by HIFs that express any protein due to fluorescent probe, and nitroreductase (NTR) based probe under hypoxia), and **(c)** demonstrating the relation between pO_2_ and sO_2_ to correlate between physiological hemodynamic-based direct and indirect methods. CBP: CREB-binding protein, HIF: hypoxia inducible factors, HRE: hypoxia-responsive element, MrO_2_: metabolic rate of oxygen, PIMO: pimonidazole, pO_2_: partial pressure of oxygen, Ru: rubidium, sO_2:_ oxygen saturation concentration.

Development and approval of a method which can accurately investigate and assess tumor hypoxia will lead to more appropriate treatment plans - which will ultimately lead to greater survival rates and reductions in cancer metastases. Existing investigations of tumor hypoxia can be categorized into two approaches: (1) determination of proteomic and genomic transformation of tumor cells in response to hypoxia, and (2) evaluation of physiological hemodynamic and/or metabolic alterations due to the onset of hypoxia (see Fig. 2).

To determine tumor hypoxia from molecular markers, we can measure the concentration of the endogenous marker HIF. HIF-1α is critical for the expression of multiple hypoxia-related targets. Under normal oxygen conditions, HIF-1α is deactivated. In hypoxic conditions HIF-1α accumulates in the cell, leading to up-regulation of multiple HIF-1α target genes 9, 13 and initiating multiple hypoxia-derived molecular processes. These processes include binding to hypoxia-responsive elements (HREs) of hypoxia-regulated genes that drive tumor growth, proliferation, and metastatic potential. Each of the HIF-1α-regulated proteins and transcription factors may be considered as a biomarker for tumor hypoxia that may actuate the metastatic process. Hypoxic tumor cell mechanisms of survival and growth depend on angiogenesis which is related to vascular endothelial growth factor (VEGF) concentration; under hypoxic conditions, glucose transporter 1 (GLUT-1) inhibits the aerobic metabolism through up-regulation to satisfy elevated glucose consumption due to anaerobic glycolysis; tumor cell proliferation and survival is regulated via nuclear factor kappa-light-chain-enhancer of activated B cells (NF-kB); decreased apoptotic potential is up-regulated by signal transducer and activator of transcription 3 (STAT3, p53) in hypoxic tumor cells; OPN binding to cell surface receptors on tumor cells activates integrins and MMP signaling pathways, increasing the tendency of tumor cell invasion, adhesion, and migration. These biomarkers are increased in hypoxic conditions, however, it has been shown that they are not tumor specific. Local upregulation of these biomarkers may indicate other physiological changes 13. However, regulation of each of these hypoxia responsive endogenous biomarkers are indicative of unique pathways of tumor progression. Therefore, contrast agents specific to these biomarkers would provide insight into the relationship between tumor hypoxia and tumor growth.

Next we consider determining and assessing tumor hypoxia through evaluation of local physiological hemodynamic and/or metabolic alterations that can be analyzed through different technologies: determining partial pressure of oxygen (pO_2_), concentration of endogenous chromophores (i.e. oxy- and deoxyhemoglobin), tissue perfusion based on blood flow measurement, oxygen saturation concentration (sO_2_), metabolic rate of oxygenation (MrO_2_) and uptake of exogenous biomarkers or radiolabeled hypoxia reporters 9, 14.

Values of pO_2_ and sO_2_ below certain threshold levels directly correspond to hypoxia. The direct method of measuring pO_2_ is using polarographic electrode-based probes 9, 14. This is also considered the 'gold standard' for measuring tumor hypoxia in a clinical setting. Although an absolute value of pO_2_ can be measured, different threshold levels have been considered in the literature for defining the onset of hypoxia. In many cases, the measured values were represented in terms of hypoxic fraction (the ratio of the number of measurements below threshold and total number of measurements). Moreover, the probe is invasive and is limited to surface tumors 9, 14. Additionally, oxygen electrode probes have been shown to give incorrect results 9, for example, in cases where the patient was administered halogenated anesthetics 8. Moreover, necrosis in the tumor tissue will cause the probe to give misleading readings 9. Another direct, minimally invasive method, phosphorescence quenching, is based on the quantification of the interaction between oxygen molecules with phosphorescent dyes 9. Quantitative pO_2_ values can also be determined by using oxygen-sensitive electron paramagnetic resonance (EPR) probes where spectral band corresponding to the probe signal correlates with oxygen concentration 9. ^19^F-magnetic resonance spectroscopy (MRS) and Overhauser-enhanced MRI (OMRI) are two magnetic resonance-based imaging modalities capable of providing absolute pO_2_.

There are several imaging modalities used to study tumor hypoxia based on the evaluation of indirect hemodynamic changes. There are two types of MRI that have been developed for indirect assessment of tumor hypoxia, dynamic contrast enhanced-MRI (DCE-MRI) and blood oxygenation level-dependent MRI (BOLD-MRI) 15, 16. DCE-MRI relies on contrast agent uptake, which is indicative of tumor perfusion, and linked to oxygenation levels 15, 16. Researchers have examined hypoxia in melanoma via this MRI technique using gadolinium-based contrast. As a result, pharmacokinetic analysis showed the fractional distribution volume of gadolinium were correlated to the hypoxia status of tumors 15, 16. DCE-MRI may also be used with manganese dioxide (MnO_2_) nanoparticles to enhance contrast between healthy and malignant tumor tissue 17. MnO_2_ can adequately diagnose hypoxic regions of tumor, may enable predictions of responses to radiotherapy and immune checkpoint inhibitors 17-21, and can aid in photodynamic therapy targeting and activation 18, 19, 22. This effect can be amplified by utilizing engineered nanoparticles that disintegrate in acidic tumor environments, for example by using calcium phosphate shells 22, 23. Moreover, studies have also loaded iron-oxide nanoparticles onto inorganic exosomes which has adequately imaged hypoxia via MRI 24-26. These agents used in DCE-MRI (e.g., gadolinium, MnO_2_ and loaded exosomes) are severely toxic for patients, so far limiting this method to research studies 27. BOLD-MRI allows one to utilize the paramagnetic properties of deoxygenated and oxygenated hemoglobin in which the weighted imaging details change in oxygenation 28. While there are many studies detailing the potential clinical applications of BOLD-MRI in understanding tumor hypoxia there are many limitations of this method as BOLD-MRI only provides qualitative information not quantitative details 28 which is crucial in understanding hypoxia.

Modalities such as positron emission tomography (PET) are minimally invasive techniques based on the uptake of intravenously administered radiolabeled reporters to detect tumor hypoxia. However, PET is not effective for determining hypoxia in a clinical setting due to impractical temporal resolution that does not allow real time imaging of tissue hypoxia 9. Moreover, it exposes patients to ionizing radiation. Near Infrared Spectroscopy (NIRS) relies on the different absorption spectra of hemoglobin (Hb) and oxyhemoglobin (HbO_2_) to quantify a ratio of Hb/HbO_2._ Based on the differential feedback of these two values, sO_2_ is calculated and an estimated pO_2_ value can be determined based on the oxygen dissociation curve. However, due to the inherent optical diffusion limit, this method provides low spatial resolution, and has a poor depth penetration 9. Indirect hypoxia assessment has also been performed based on contrast agents binding to endogenous chromophores or immunohistochemical (IHC) staining of exogenous reporters. *In vivo* bioluminescence imaging 29-32, where chemical energy is transformed into light energy and photons emitted by cells/tissues are detected 29, is based on luciferase activity, luciferin and oxygen 29-32. This imaging method has been used to investigate breast, ovarian, prostate and lung cancers 33-37. This method uses the transcription factor HIF-1α to approximate the degree of hypoxia 29, 30, 32. While this method shows some promise in investigating tumor hypoxia, there are many limitations: it does not provide direct information on oxygenation levels 29, 30, 32 and studies have emphasized to use caution when using this method as it can underestimate tumor hypoxia 32. The detection of pimonidazole (PIMO) and pentafluorinated etanidazole (EF5) accumulation, two lipophilic exogenous hypoxia markers, in hypoxic tumor using immunohistochemical techniques or flow cytometry also provide estimation of the pO_2_ 38. However, these approaches are complex.

Fluorescent microscopy is an established imaging modality and a plethora of fluorescent probes able to detect hypoxia through bioactivity of nitroreductase (NTR) and other mechanisms exist. The difficulty of using fluorescent microscopy lies with the poor penetration of optical microscopy in general, requiring highly luminescent signals to penetrate through highly attenuating biological tissue. For example, the probe GCTPOC-HY has a 130-fold fluorescent enhancement, yet when applied to monitor NTR via two-photon microscopy it only demonstrated a penetration depth of 70 μm 38. Near infrared fluorophores conjugated to hypoxia reporters can extend penetration depth and their use for tumor detection in mice has been reported 39.

Table 1 summarizes the available modalities, clinical acceptability status, and their functionality towards investigating tumor hypoxia. There are a multitude of methods at our disposal to better understand tumor hypoxia. However, the limitations associated with these methods demonstrate a need for a different method that is non-invasive and capable of utilizing both the genomic and physiologic approaches for investigating tumor hypoxia. PAI, a combination of optical and ultrasound imaging has great potential to overcome the existing challenges and limitations seen by other methods.

Photoacoustic imaging (PAI) is an emerging technique which couples optical sensitivity with acoustic detection capabilities and has been used extensively in research 41-54. Researchers have explored PAI for a number of clinical uses 55, including quantification of risk from acute pneumonia to persons with atherosclerotic plaque 56, risk of drug-induced hepatotoxicity 57, 58, estimation of brain oxygenation via jugular venous oxygenation 59 and impairment of heart function 60. Another major application of PA imaging is its capability using either endogenous or exogenous contrast agents to analyze tumor hypoxia. In this review, we principally discuss articles which investigate tumor hypoxia via PA imaging. Initially, we explain the working principles of PAI and technological advantages. Next, we categorize photoacoustic (PA) approaches to detect hypoxia. Methods involving label free imaging, as well as organic and inorganic contrast agents, are included in the categorization that encompasses both the genomic transformation and physiological/ metabolic evaluation approaches. The benefits and limitations of these categories from an application and clinical standpoint are also assessed. Developing an in-depth understanding of these PAI approaches to study tumor hypoxia will help create a more comprehensive understanding of what currently can be done with this imaging modality, as well as create an opportunity to better understand what possible future applications can be achieved in clinical and research settings.

## 2. Basic Principles of PAI and its Importance in Assessing Tumor Hypoxia

### 2.1 Principles of PAI

In PAI, the imaging target (optical energy absorber) is optically excited using a pulsed laser, leading to a transient temperature increase and resulting in a thermoelastic expansion of the absorber followed by emission of acoustic pressure waves. This initial pressure rise 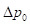 following light absorption can be written as:


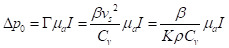

(1)

Where the Grüneisen coefficient, 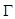 can be represented by the thermal expansion coefficient (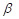), the compressibility (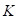), and the speed of sound (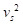) and can further be expanded in terms of the mass density (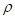) and specific heat capacity at constant volume (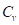). The absorbed optical energy is represented as the product of the absorption coefficient (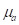) and the fluence at the local absorption point (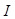). The emitted acoustic pressure waves from the absorber are propagated through the adjacent tissue layers and eventually detected by ultrasound (US) transducers placed on the skin. These acoustic waves induced from optical energy absorption, also known as PAsignal, are used to generate real-time, high-resolution images representing functional and molecular characteristic distributions of tissue chromophores at higher depths than pure optical imaging. Therefore, PAI combines the advantages of the unique optical absorption characteristics of tissue chromophores with the higher penetration depth of ultrasound 61-67.

### 2.2 Use of PAI for directly measuring endogenous markers

The physiological and pathological status of the tumor (i.e., vascular pattern, angiogenesis, necrosis) can be well defined based on the intensity contrast (compared to background) in PA images generated by the absorbing chromophores within and around the tumor region 68, 69. Moreover, the changes in optical absorption as a function of wavelength for a variety of endogenous chromophores (i.e., melanin, collagen, oxy- (HbO_2_) and deoxy-hemoglobin (HbR)) enables spectroscopic or multi-wavelength imaging techniques to denote functional characteristics of the tumor region 70. For example, HbO_2_ and HbR have different wavelength-dependent optical absorption properties, which allows spectroscopic PAI to differentiate between arteries and veins. Furthermore, optical absorption by HbO_2_ and HbR at two or more wavelengths (including the same optical absorption at ~758 nm), can be used to assess total hemoglobin concentration (tHb) and, therefore, derive, blood oxygen saturation (sO_2_) 61-63. Based on the linear spectral fitting model 71-73, this is achievable as the absorption coefficients (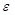) of HbO_2_ and HbR are known at specific wavelengths and HbO_2_ and HbR concentrations (*C_HbO_*_2_ and *C_Hb_*) at specific image locations can be determined from PA signal intensity. Total Hb can be calculated by 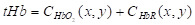, and sO_2_ can be calculated as follows:


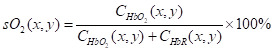

(2)

The hemoglobin-based oxygen saturation measurement is referred to as “label-free” PAI 61-63. Compared to other blood oxygenation imaging modalities, label-free PAI exhibits several advantages. In comparing functional magnetic resonance imaging (fMRI) to PAI, PAI allows noninvasive *in vivo* oxygenation imaging at a relatively low cost 74-78. Unlike PET, PAI uses nonionizing laser illumination and provides a much higher spatial resolution. Lastly, the induced acoustic wave in PAI is less attenuated as compared to strong optical scattering in pure optical imaging in biological tissue allowing the PA signal to propagate a greater distance than photons without losing their original propagation direction.

In 14, MrO_2_ has been successfully quantified from PA imaging based on the following equation:


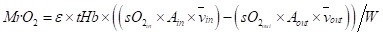

(3)

Where *ε* denotes the oxygen binding capacity of hemoglobin and is usually considered a constant, *A* denotes cross-sectional area, 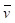 is the average blood flow speed (*in* or *out*), and *W* is the weight of the region of interest. These parameters are required to be measured locally. Anatomic parameters i.e., weight and area are quantified from the structural information extracted from PA image and fluid-dynamic parameter i.e., flow speed can be estimated on the basis of PA Doppler bandwidth broadening of the PA signal induced by circulating red blood cells. Another physiological assessment of hypoxia can be implemented by measuring oxygen extraction factor (OEF) as follows 79:


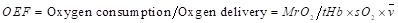

(4)

### 2.3 PA signal enhancement with exogenous markers

PA contrast can also be enhanced by exogenous contrast agents (organic or inorganic) that have distinct and tunable absorption spectra in the near-infrared (NIR) optical window (600-1100 nm) 61-63, high oxygen sensitivity, and endogenous protein binding specificity. Furthermore, excitation of the tumor region where these agents are bound enhances spatial resolution, specifically when the detected PA intensity from the tumor site is too low due to very low concentration of endogenous contrast agents 61-63. Exogenous contrast agents can be used to estimate pO_2_ in tissue 61-63. This is calculated by using a time dependent correlation of PA amplitude decay that corresponds to the half-life of the contrast agent. This technique uses two lasers pulsed in sequence; the first pulse excites the contrast agent forming a transient molecule, which is then excited by the second laser pulse to generate PA signals. The time delay between the two laser pulses is tuned to optimize the PA amplitude. In areas with high pO_2_ concentration, the contrast agent half-life is reduced, decreasing the PA signal intensity. An exponential fitting function is applied to the PA signal intensity decay to calculate pO_2_ 63. Unlike conventional spectral-based methods that give only oxygen saturation levels, PAI can yield simultaneous 3D imaging information including anatomical, functional, histological, and metabolic parameters.

Although PAI has significant benefits in a research setting, realization of this modality is also practical in a clinical setting 77 due to easy integration with existing ultrasound technology, low cost and minimal to non-invasive nature. All of these factors make PAI a promising clinical tool for investigating tumor hypoxia 74, 80-82.

## 3. Review Method

Here, we reviewed various approaches for evaluating biomarkers of tissue hypoxia, such as oxygen saturation, partial pressure of oxygen, and metabolic rate of oxygen consumption. The assessments of hypoxia approaches were classified as follows: (1) from physiological changes using endogenous, label-free biomarkers; and (2) from physiological/ molecular alterations using exogenous contrast agents by either (a) an estimation of physiological changes using exogenous contrast agents or (b) an estimation of oxygen sensitive proteomic transformation/exogenous contrast agent activation. Journal articles relevant to this review were gathered by conducting an online search using PubMed. The goal of this review is to study relevant articles which used PAI to assess tumor hypoxia. This initial search utilized the keywords: 'photoacoustic imaging', 'hypoxia', 'tumor hypoxia' and 'photoacoustic technologies'. Furthermore, the following MeSH terms were also used: 'contrast agents', 'hypoxia' and 'cancer'. This search yielded 174 results, of which 26 papers were selected for this review. The inclusion criteria for conducting the review were the articles which utilized *in vivo* or *in vitro* experiments, utilized PAI specifically as well utilized animal cancer models. The exclusion criteria of this paper were articles which utilized PAI to determine the efficacy of hypoxia targeted drug delivery, papers which used modalities aside from PAI to investigate tumor hypoxia such as bioluminescence imaging, fluorescence imaging, and PET.

## 4. Assessment of hypoxia from physiological changes using endogenous (label free) biomarkers

**Figure 3:**
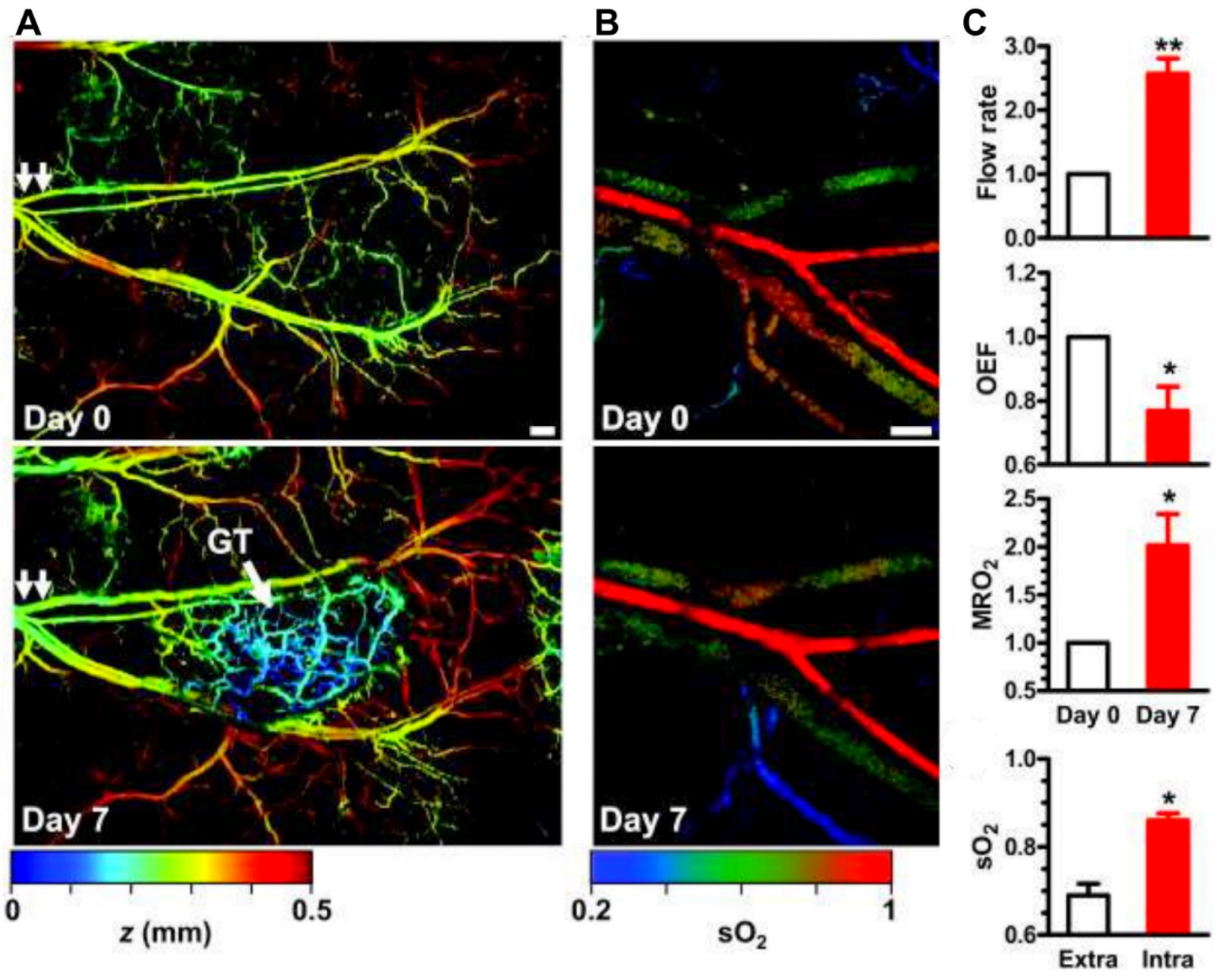
**Metabolic PAmicroscopy (mPAM) detection of early-stage glioblastoma using MRO_2_. (a)** mPAM images of microvasculature in the tumor region at 584 nm. Depth (*z*) is coded by colors: blue (superficial) to red (deep). Scale bar: 250 μm. The arrow labelled GT is indicative of the glioblastoma tumor, the double arrows in both panels is the location from which tumor sample was taken for additional experiments in this study, **(b)** mPAM images of sO_2_ in the artery-vein pair [double arrows in (b)] that supports the tumor region acquired on day 0 and day 7. Scale bar: 100 μm, and **(c)** mPAM quantification of volumetric blood flow rate, OEF, MrO_2,_ and averaged sO_2_ seven days after the tumor xenotransplantation, normalized by the values of day zero. OEF: oxygen extraction factor, MrO2 MrO_2_: metabolic rate of oxygen, sO_2_: oxygen saturation concentration. Adapted with permission from 14, copyright 2011, SPIE.

Label free imaging provides the advantage of using the unique absorption spectra of endogenous contrast agents, particularly, oxygenated and deoxygenated hemoglobin 83, 84. This unique property is well known, and most of the PAI systems use specific wavelengths such as 750 nm and 850 nm to correlate to oxygenated and deoxygenated hemoglobin respectively for tumor hypoxia study 85. However, other wavelengths may also be used to measure oxygenated and deoxygenated hemoglobin. For example, a study utilized excitation wavelengths at 584 nm and 590 nm 14. From these wavelength specific parameters, blood oxygen saturation (sO_2_) values were determined as explained in section 2. Label free imaging has been shown to adequately measure oxygenation in 14, 86-88. Specifically, sO_2_, and MrO_2_ have been determined via label free PAI 14, 86-88. For example, an animal model study utilizing metabolic PA microscopy (mPAM) has shown the OEF decreases by 43% in melanomas. Moreover, OEF decreases by 24% in glioblastomas (See Fig. 3) 14. A lower OEF value is indicative of a significant decrease in oxygen transport across the capillary wall 79. This in turn will result in a decrease of oxygenation in tumor tissues and eventually causing worse tumor hypoxia 14. This study 14 also demonstrated hypermetabolism in melanoma with a 36% increase in MrO_2_ at early stages followed by a gradual decline in MrO_2_ starting from day 14. This phenomenon explains the relative gradual tumor growth as well as tumor core necrosis.

**Figure 4:**
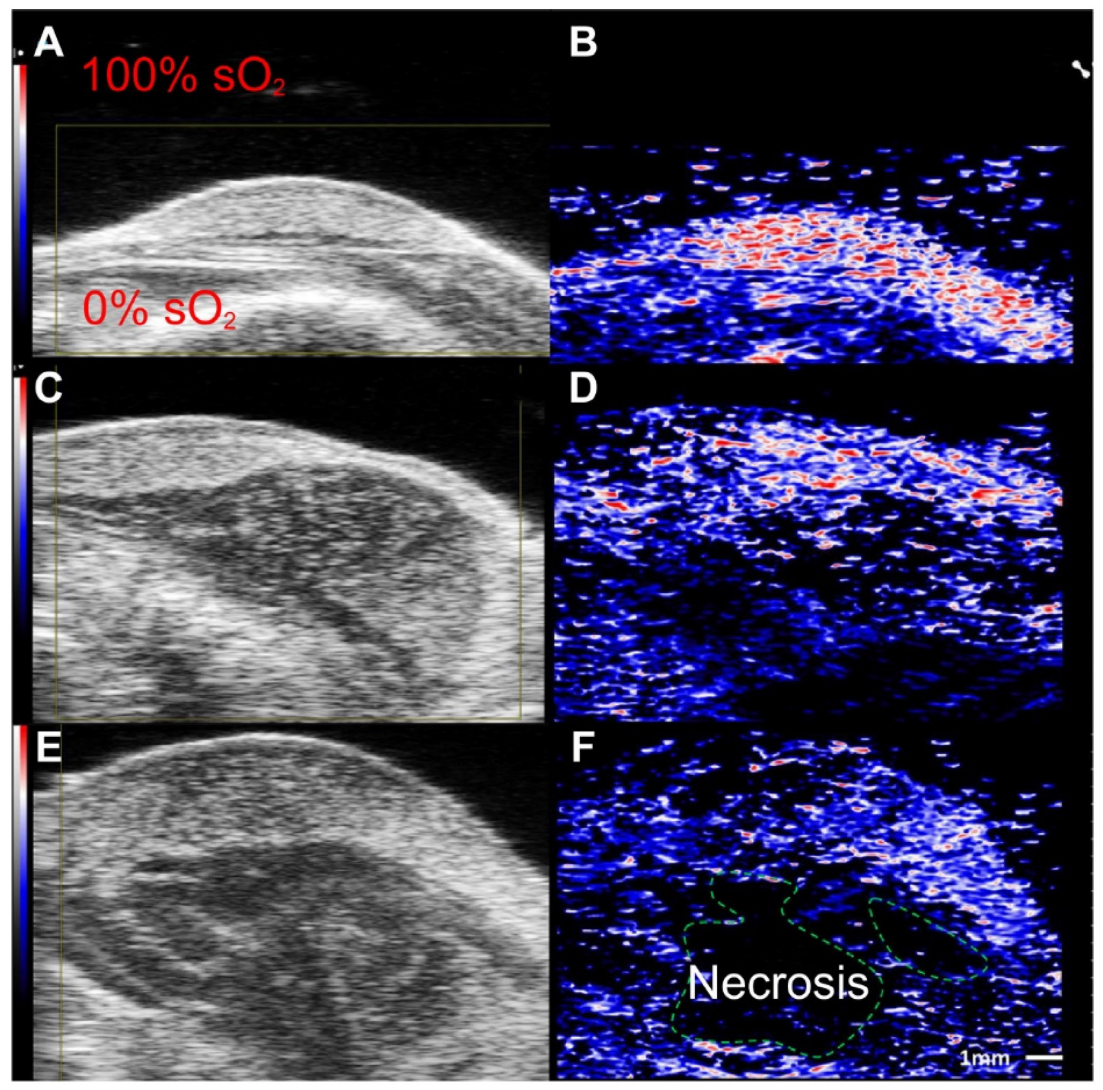
**Label free imaging of subcutaneously implanted patient derived xenograft lymphoma:** Label free imaging of mouse tumor model on ultrasound B-mode and hemoglobin based sO_2_ map from PAI. Differences in tissue architecture seen on panels a,c,e. correlate to panels b,d,f in which brighter pixels (red and blue) indicate oxygenated areas. Areas devoid of oxygen show darker regions in PAI and are thus necrotic zones. sO_2_: oxygen saturation concentration. Reproduced from Ref. 86, copyright 2021, Oxford.

Moreover, label free imaging has been able to document changes in local oxygen saturation as a tumor progresses 89. Researchers utilized PAI to assess pancreatic tumors in an animal model, in which oxygenation levels of the tumor were compared at 7 and 21 days post implantation. At day 7, sporadic hypoxic regions were found. However, at day 21, there was a significant decrease of oxygenation levels, and a statistically significant increase of deoxygenated hemoglobin 89. Furthermore, studies using label free imaging have shown sO_2_ levels are inversely correlated to tumor volume 86 (See Fig. 4) where, tumor volume increases as the sO_2_ levels decrease 87. In addition, label free imaging has been used to detail the alterations in sO_2_ as the tumor grows 87, 88 which demonstrates that label free imaging can be a tool to monitor changes in tumor hypoxia 79 and size.

Label free imaging was used to investigate tumor hypoxia in multiple types of cancers 14, 86-88, 90. This method has also been effective in assessing the distribution of oxygen within a solid tumor, in which label free PAI has detailed the tumor core to be hypoxic, whereas the 'rim' of the tumor was well oxygenated. The consensus arrived at from these label free studies is tumor tissue has oxygenation levels that are measurably different from healthy tissue 14, 86-88 and higher fluctuations are seen in oxygen saturation in tumor models with higher disease aggressiveness 90. Label free imaging has also been shown to be responsive to changes in tumor oxygenation upon external application of oxygen 14. This is pertinent as it shows label free imaging may be a way to potentially determine the efficacy of different cancer therapies targeted towards hypoxia.

In addition, label free imaging can potentially be used as a cancer risk assessment tool 91. When testing for cervical cancer, typically vaginal mucosa samples from pap smears are assessed. While this has been an effective method and is widely used clinically, it does not show a positive test result when the cancer is microinvasive. To mitigate this, an endoscopic system with a compact optical based fiber was designed, in which dual mode light delivery was achieved. PA images were taken of tissue phantoms imitating cervical tissue which adequately showed changes in the spatial relationship between hemoglobin concentration, oxygenation, and blood perfusion. Therefore, this system can potentially increase the efficiency of cervical biomarker detection, which opens the door for clinical applications of PAI 91.

Furthermore, label free imaging may be useful in predicting tumor recurrence. Murine model of glioblastoma implanted in hind leg, receiving photodynamic therapy (PDT), was imaged with label free PAI to determine treatment efficacy and risk of tumor recurrence 92. Interestingly, it was found that models responding to the therapy have an increase of up to 85% in skeletal muscle oxygen saturation levels. Skeletal muscle oxygen saturation levels can be mapped and tumor recurrence risk can be assessed within 24 hours, based on the efficacy of the therapy. This is remarkable, since, tumor recurrence risk typically cannot be evaluated until 10-30 days post treatment 92.

**Figure 5:**
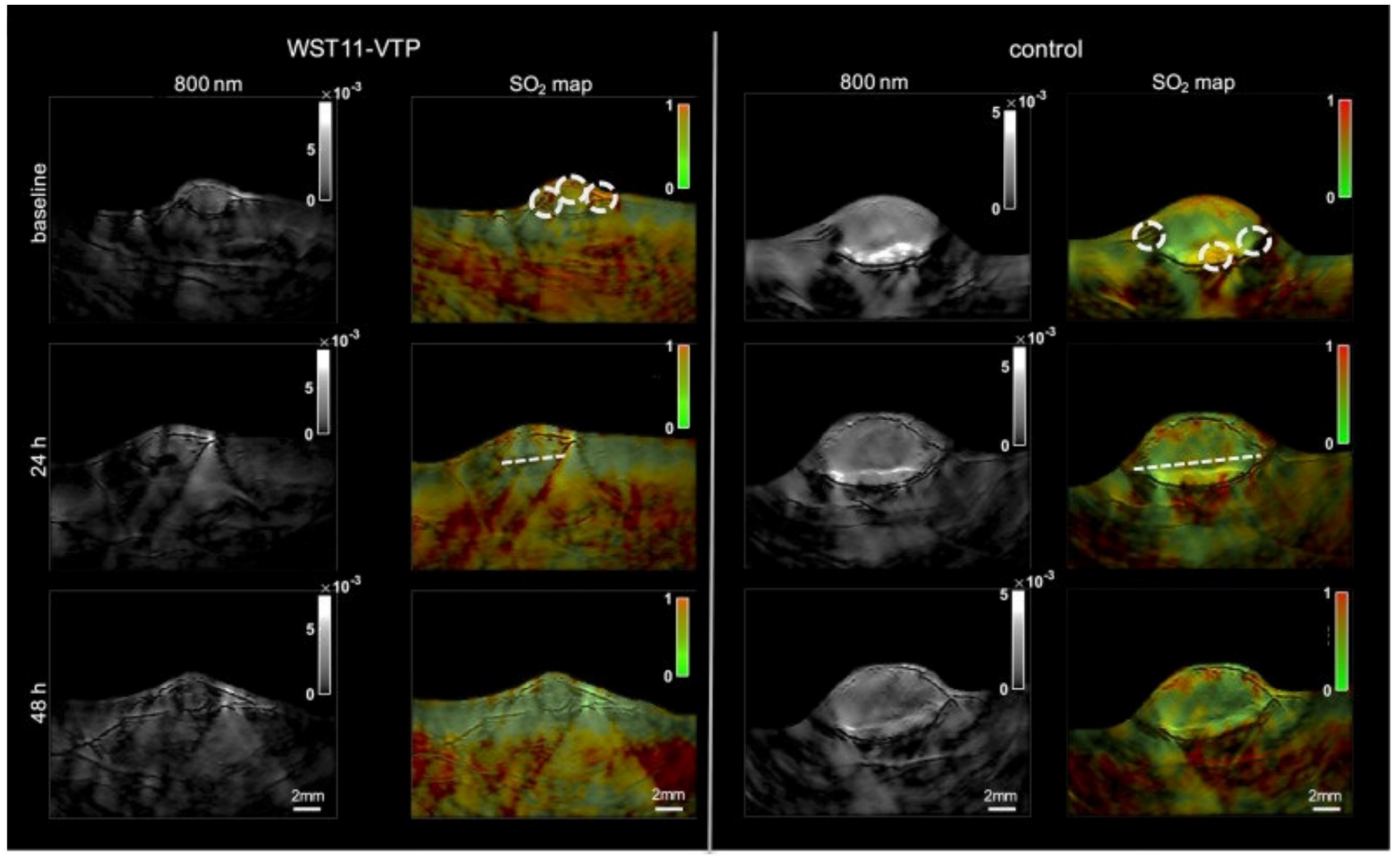
**Treatment effect monitoring over 48 h by multi-spectral optoacoustic tomography (MSOT).** MSOT was found to be able to monitor the oxygen saturation within tumor tissue as a critical parameter leading to tumor tissue destruction induced by WST11-VTP over the course of 48 h. In comparison, the control mice of saline-VTP sham treatment showed only very limited signs of reduced oxygen saturation within the tumor region of interest. sO_2_: oxygen saturation concentration, VTP: vascular targeted photodynamic therapy. Reproduced from 93, copyright 2018.

Moreover, another research group utilized multi-spectral optoacoustic tomography (MSOT) (functional PA tomography) to determine the impact of vascular targeted photodynamic therapy on sO_2_ 93. In this study, renal cell cancer mouse models were used, and an sO_2_ map was generated pre- and post-PDT using MSOT. Interestingly, 40 minutes post-PDT the entire tumor center became hypoxic, and 60 minutes post-PDT the whole tumor mass was hypoxic showing a 60% drop in sO_2_. However, oxygenation levels returned to baseline 48 hours post-PDT, whereas the control group showed consistent sO_2_ levels over the course of 48 hours (see Fig. 5). These studies are impactful as they show PAI can be used in a clinical setting to determine treatment efficacy as well as the impact of cancer therapies on oxygenation over a long period of time.

In addition to simply reporting oxygenation levels, PAI can be used to report how oxygenation levels change in response to particular, targeted therapies. For example, PAI has been used to monitor intra-tumoral oxygen saturation changes caused by release of catalase and 5-ALA co-loaded nanoparticles 94. Another study monitored the response of nanozyme and glucose oxidase by analyzing PA signal variation 95.

Label free imaging can be used to monitor changes in tumor hypoxia during a course of treatment. While label free imaging is a superior method to ionizing methods, the amount of information provided by label-free imaging is still limited. In an animal model study where liver metastasis was induced via adenocarcinoma cells, label free imaging confirmed the decrease in overall sO_2_ levels 88. However, label free imaging was ineffective in determining the degree of the disease stage and could not accurately determine the likelihood of a metastases 88.

## 5. Assessment of Hypoxia from physiological/molecular alterations using exogenous contrast agents

**Figure 6:**
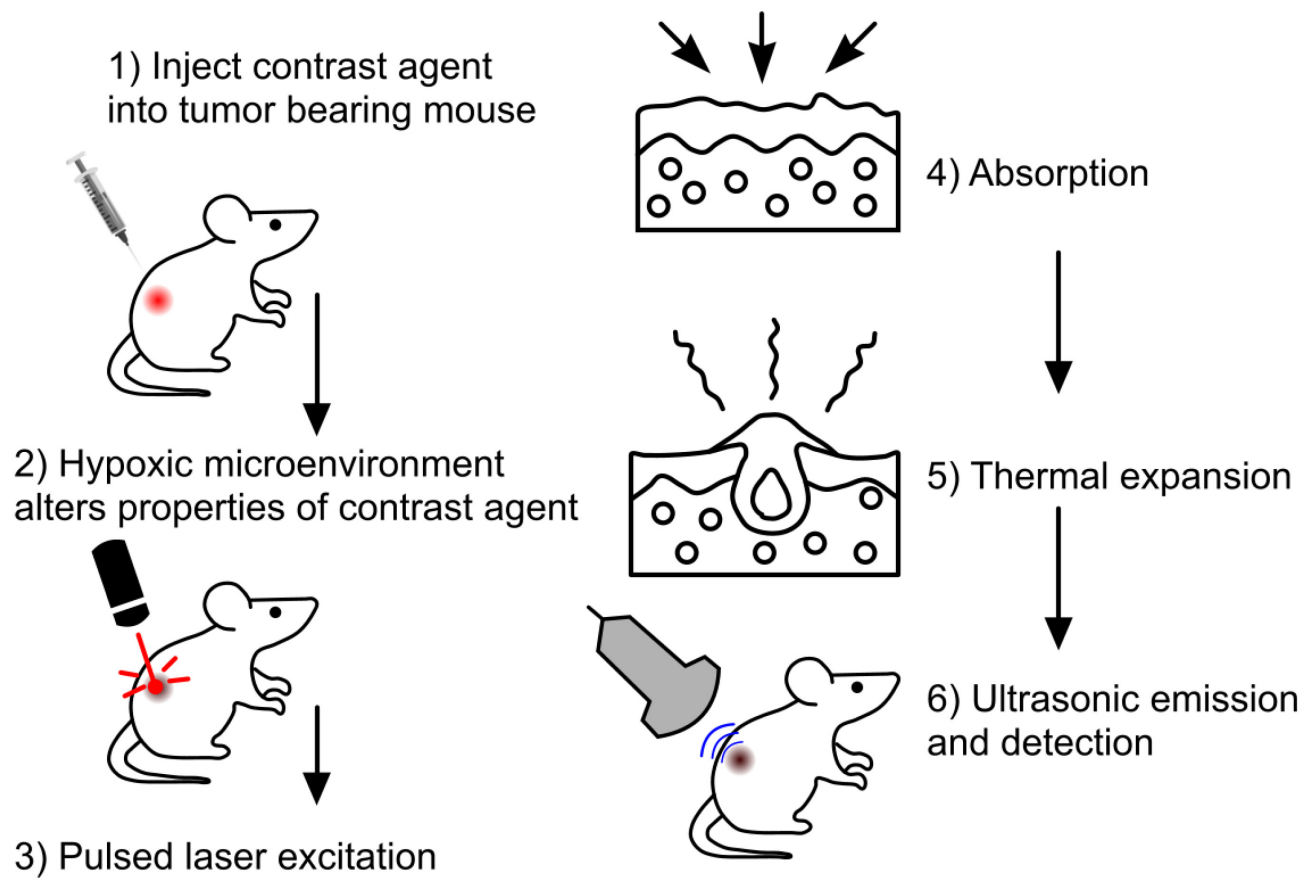
**General steps in imaging with exogenous contrast agents** Step 1: initially tumor bearing mice were injected with the exogenous contrast agent, e.g., dyes or nanoparticles. Step 2: Upon injection the agent will bind or be modified to provide better PA signaling. Step 3: Laser excitation allows Step 4: the absorption of optical energy and followed by Step 5: thermal expansion. Step 6: Leads to detectable PA waves being generated, allowing image formation to occur.

The employment of exogenous contrast agents can mitigate some of the shortcomings of label free imaging, as well as amplify detection accuracy 84. These agents can provide higher depth penetration, sensitivity and specificity, as well as a better signal-to-noise ratio in comparison to endogenous agents like hemoglobin 96. This is attributed to these contrast agents having a stronger NIR absorbance and better photothermal conversion than endogenous agents 96. General steps of PAI using exogenous contrast agents are depicted in Fig. 6. Exogenous contrast agents can be classified as organic and inorganic. Organic contrast agents can consist of polymer-based nanomaterials and dyes 38. Polymer-based nanomaterials are used for *in vivo* bioluminescence imaging however, not for tumor hypoxia. Dyes are typically porphyrin and cyanide based 47. These dyes show a great degree of biocompatibility and biodegradability 38. Furthermore, these dyes are small enough to cross the blood brain barrier 38. In addition, cyanide-based dyes have been approved by the FDA 38, amplifying, the potential clinical applications of PAI. While there are benefits to utilizing dyes in PAI, there are a multitude of shortcomings as well. Dyes tend to have a short circulation half-life, limiting the imaging duration. Moreover, dyes tend to show poor photothermal stability and poor aqueous solubility 38, as compared to inorganic contrast agents. Carbon based nanomaterials are another type of organic contrast agent which can assess tumor hypoxia. A study used *hypocrella bambusae* prepared with carbon dots 97. *Hypocrella bambusae* can generate oxygen and has a high red light emission maximum. When combined with carbon dots, the complex is referred to as HBCDs. PAI was used to determine the efficacy of this agents' accumulation in the tumor. Upon irradiation at 635 nm, PA images showed clear tumor microstructure, demonstrating this agent may be an effective tool in photoacoustic-guided tumor therapy 97.

The other category of exogenous contrast agents is inorganic 38, 96. These agents can be subdivided into three further categories: (1) metallic nanomaterials, (2) polymer-based nanomaterials, and (3) transition metal chalcogenides (TMC) 38. Examples of metallic nanomaterials include gold nanorods or nanocages, and palladium nanoplates or antimony particles 38. Some of the benefits of using metallic nanomaterials is their tunable optical properties, and good biocompatibility 38. Nanoparticles (specifically gold) are considered to be better than molecular markers, as they have high fluorescence quenching from their unique surface plasmons 98.

### 5.1 Estimation of physiological changes using exogenous contrast agents

To estimate physiological changes, organic dye methylene blue (MB) has been implemented due to its oxygen sensitivity 99 with two laser method (laser 1 absorption peak at 650 nm and laser 2 excited state peak at 810 nm) of pO_2_ extraction. In the presence of oxygen, the dye is activated and turned blue, thus eliciting 'oxygen sensitive' properties. MB has been shown to adequately identify hypoxic regions in pancreatic tumor bearing mice 99 where hypoxic regions were differentiated based on pO_2_ at 650 nm (hypoxic areas) and 810 nm (well oxygenated areas).

**Figure 8:**
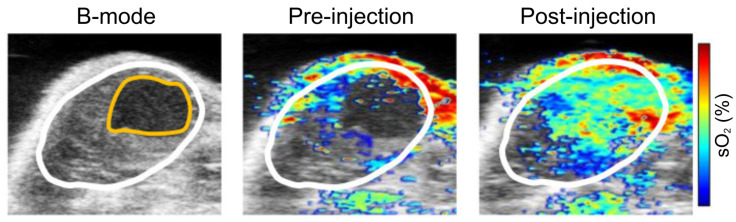
**Nanocrystals were conjugated with MnO_2_ shells for sO_2_ measurement using PAI.** sO_2_: oxygen saturation concentration. Adapted with permission from 100. sO_2_: oxygen saturation concentration, copyright 2020, MDPI.

Inorganic exogenous contrast agents were mostly utilized through binding with endogenous chromophores/ proteins. Nanoparticles formed by enclosing nanocrystals with manganese dioxide (MnO_2_) have been a popular exogenous contrast agent 100 due to their ability to form hybrid complexes. As tumor hypoxia renders an increased production of reactive oxygen species (ROS), as well as a more acidic environment, this contrast agent will react with ROS to create H_2_O and O_2_ and re-oxygenate the area. When testing the efficacy of this contrast agent, PAI was employed and showed poorly oxygenated areas were reoxygenated (increased sO_2_, see Fig. 8). Thus, showing this nanoparticle is effective in reacting with and distinctly finding hypoxic regions of the tumor 100. Mice with the targeted contrast agent had a significantly increased signal for HbO_2_.

Many studies used metallic nanomaterial and PAI to guide radio/chemotherapy 22, 101, 102. For example, polyacrylic acid-coated superparamagnetic iron oxide nanoparticles were modified to be cisplatin-loaded, poly-dopamine coated and GE11 peptide coated, creating GE11-PDA-Pt@USPIOS. One of the goals of this contrast agent was to catalyze the decomposition of H_2_O_2_ and make oxygen, thus re-oxygenating the tumor. To demonstrate the oxygenation status after injection of nanoparticles, PAI was performed at 800 nm 101.

Another attempt to assess the effectiveness of PAI based on sO_2_ evaluation involved polymer-based inorganic contrast agents which were doxorubicin-loaded, hypoxia dissociable nanoparticles 103. These nanoparticles consist of self-assembled polyethyleneimine-nitroimidazole micelles. These were co-assembled with hyaluronic acid-Ce6 to develop the inorganic contrast agent called: HC/PN/DOX-NP that enters tumor cells via CD44 cell endocytosis. Researchers used 660 nm optical illumination to demonstrate decrease in sO_2_ levels from 15.5% to 2.1%. which showed that mice treated with the contrast agent had inhibited tumor growth 103.

### 5.2 Estimation of oxygen sensitive proteomic transformation or exogenous contrast agent activation

**Figure 9:**
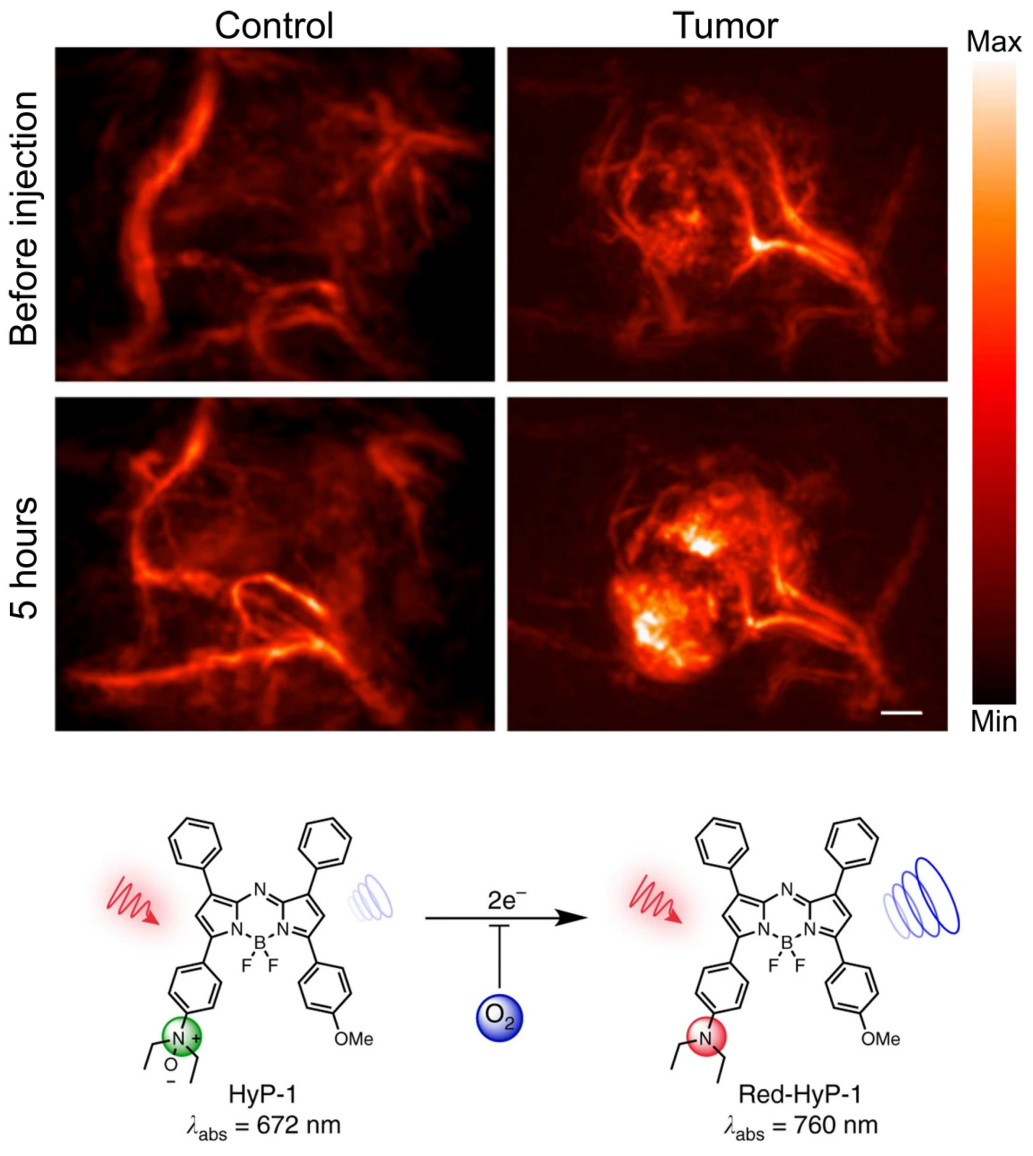
**PAI of murine hindlimb ischemia model injected with HyP-1:** PA images taken at 770nm. Changes in signal intensity pre-injection and 1-hour post-injection. Scale bar represents 2mm. The limited time frame between induction of ischemia and imaging suggests that HyP-1 does not rely significantly on upregulation of heme-based redox proteins, but rather can undergo rapid conversion to red-HyP-1 under hypoxic conditions. Molecular structures of the unreduced (low contrast) and reduced (high contrast) imaging probe are also shown. Reproduced from 104, copyright 2017 Nature.

Exogenous contrast agents have also been explored to assess hypoxia through binding to upregulated endogenous biomarkers in tumor hypoxia. Organic dye HyP-1, derived from *Hypericum perforatum*, can be used as an exogenous contrast agent in PAI, due to its peak absorbance at 670 nm 104. However, in hypoxic conditions HyP-1 will undergo a bio-reduction by heme proteins like cytochromes P450 (CYP450) and turn into 'red-HyP-1' and as a result will have an absorption at 770 nm. Hypoxia-mediated response of HyP-1 in the hindlimb ischemia model can also be observed PAI (See Fig. 9). An increase of 20.5 au in PA signal was observed in tumors exposed to HyP-1 as compared to control. Furthermore, the degree of hypoxia in distinct regions of the tumor could be extrapolated from the signal intensity. In other words, higher intensity of red-HyP-1 is indicative of increased hypoxia. Increased levels of hypoxia can be attributed to an increase risk of metastasis 104. Despite HyP-1 being an effective dye, there are always methods to make dyes better.

For example, the dye HyP-650 was derived from the properties seen in HyP-1 105. HyP-650 has an absorption maximum at 650 nm, whereas its red-shifted product has an absorption maximum at 740 nm. HyP-650 has a peak molar extinction coefficient that is twice as high as HyP-1, and a quantum yield that is ten times lower. This difference in properties makes HyP-650 a preferred contrast agent to HyP-1. Furthermore, HyP-650, has been proven to adequately measure both intra- and extravascular oxygenation levels, as has been seen in a study utilizing a breast tumor model. This is a unique finding as breast tissue has highly dynamic vasculature 105. Therefore, seeing both intra- and extravascular oxygenation values is very helpful when determining treatment plans 24. HyP-1 and HyP-650 are not the only dyes which are targets of CYP450.

**Figure 10:**
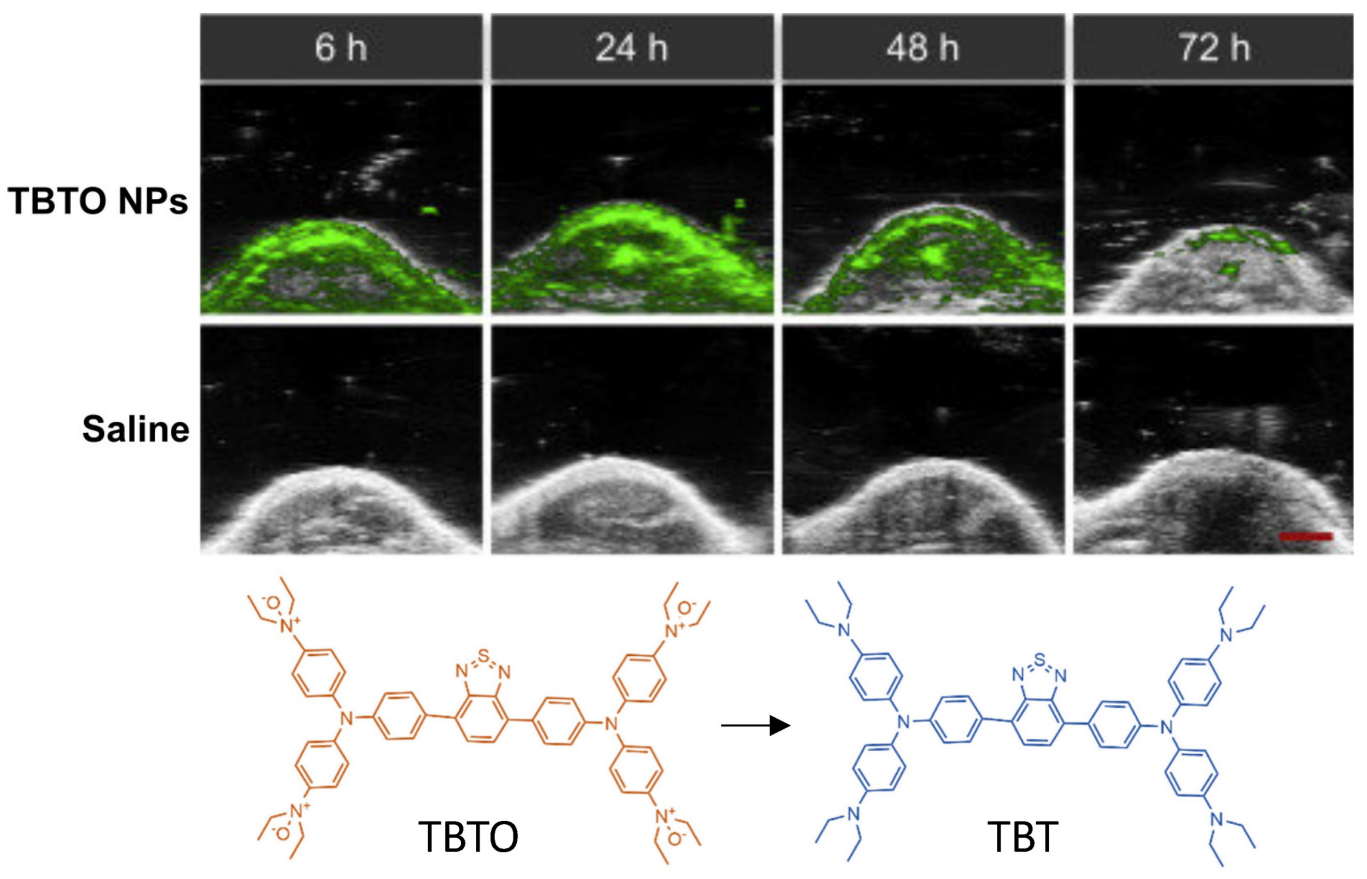
**PAI of tumor bearing injected with TBTO NPs vs control (saline)_:_** *In-vivo* PA images of tumor site of mice with injection of TBTO NPs or saline from 6h-72h. Clearance of agent was seen to occur at 72 hours. Molecular structures of the unreduced (TBTO, low contrast) and reduced (TBT, high contrast) imaging probes are also shown. NP: nanoparticle, TBT, triphenylamine-benzo thiadiazole diethylamine, TBTO: triphenylamine-benzothiadiazole diethylamino oxide. Adapted with permission from 106, copyright 2021, Cell Press.

Triphenylamine-benzo thiadiazole diethylamino oxide (TBTO) is another dye which is designed to turn into TBT (triphenylamine-benzo thiadiazole diethylamine) due to the over-produced reductases in the hypoxic tumor site 106. TBTO has an emission maximum at 600nm, and TBT has an emission maximum at 740nm 106. In an *in vitro* study, HeLa cells were implanted into mice where the heterogeneity of tumor hypoxia was visualized with PA imaging using TBTO encapsulated within pegylated lipid nanoparticles (TBTO NPs). Results are shown in Fig. 10. While the authors did not report the specific wavelength used for PA imaging, in their supplementals they demonstrated that the PA wavelength showed a strong signal for TBT NPs and an absence of signal for TBTO NPs, so it is presumed imaging was around 750 nm. Additionally, TBT persisted with a measurable degree of PA intensity for over 72 hours after injection. Thus, showing pegylated NPs containing TBTO can be a method to measure tumors over a long period of time 106. This method offers a unique application in which the efficacy of hypoxia assessment can be performed *in vitro* post administration.

There are other dyes which have similar properties to the aforementioned dyes, however, they undergo activation by different mechanisms. This can render different results as the mechanism of activation depend on different qualities seen in hypoxic environments. Dyes have been developed to take advantage of proteomic features of the tumor microenvironment. For example, NR-Azo is a dye which relies on the upregulation of proteins in tumor hypoxia 107, particularly, based on the overexpression of azoreductases which, cleave the Azo moiety in the dye. As a result, an NIR fluorophore called NR-NH_2_ is released, resulting in a peak absorption shift to 710nm from 575nm in NR-Azo. Furthermore, this dye has shown to highlight hypoxic regions *in vitro* on mice implanted with hepatocarcinoma whereas, control mice showed no expression 107.

Furthermore, cyanide-based dyes can also render a detailed view of hypoxia in tumors 108. A cyanide-based dye called As-Cy-NO_2_, utilizes a nitrobenzene moiety. This was to take advantage of the overexpression of nitroreductases in the hypoxic environment when 4-nitrobenzene moiety in this agent 108 is cleaved and As-Cy-1 is released. A study utilizing an *in vivo* xenograft breast cancer model displayed the degree of tumor hypoxia, and also adequately determined the heterogeneity of the hypoxia within the tumor 108. Similar to other dyes, this also allows the determination of hypoxia variation across a tumor* in vivo*.

Organic contrast agents can also take advantage of receptors that are upregulated in tumor hypoxia. In the presence of tumor hypoxia, the integrin α_v_β_3_ is upregulated 88 and overexpressed on endothelial cells. Moreover, the upregulation of this receptor is indicative of angiogenesis, which in turn can be indicative of hypoxia. The contrast agent Angiostamp800, binds to integrin α_v_β_3_, and generates a PA signal at 800 nm. In a study utilizing metastasized hepatocarcinoma cells in mice 88, a significant increase of Angiostamp800 signaling in tumor tissue was observed as opposed to healthy tissue. Another dye, IRDye800-NHS derived from indocyanine-green (ICG) conjugated with cyclic peptide cyclo (Lys-Arg-Gly-Asp-Phe) has also been demonstrated to target integrin α_v_β_3_ in brain tumor 71. α_v_β_3_ targeted gold nanobeacon (α_v_β_3_-GNB), an inorganic contrast agent, was also tested at 740 nm to 810 nm in blood and the authors reported a 10-fold higher PA contrast was observed as compared to the endogenous biomarkers in 109.

**Figure 11:**
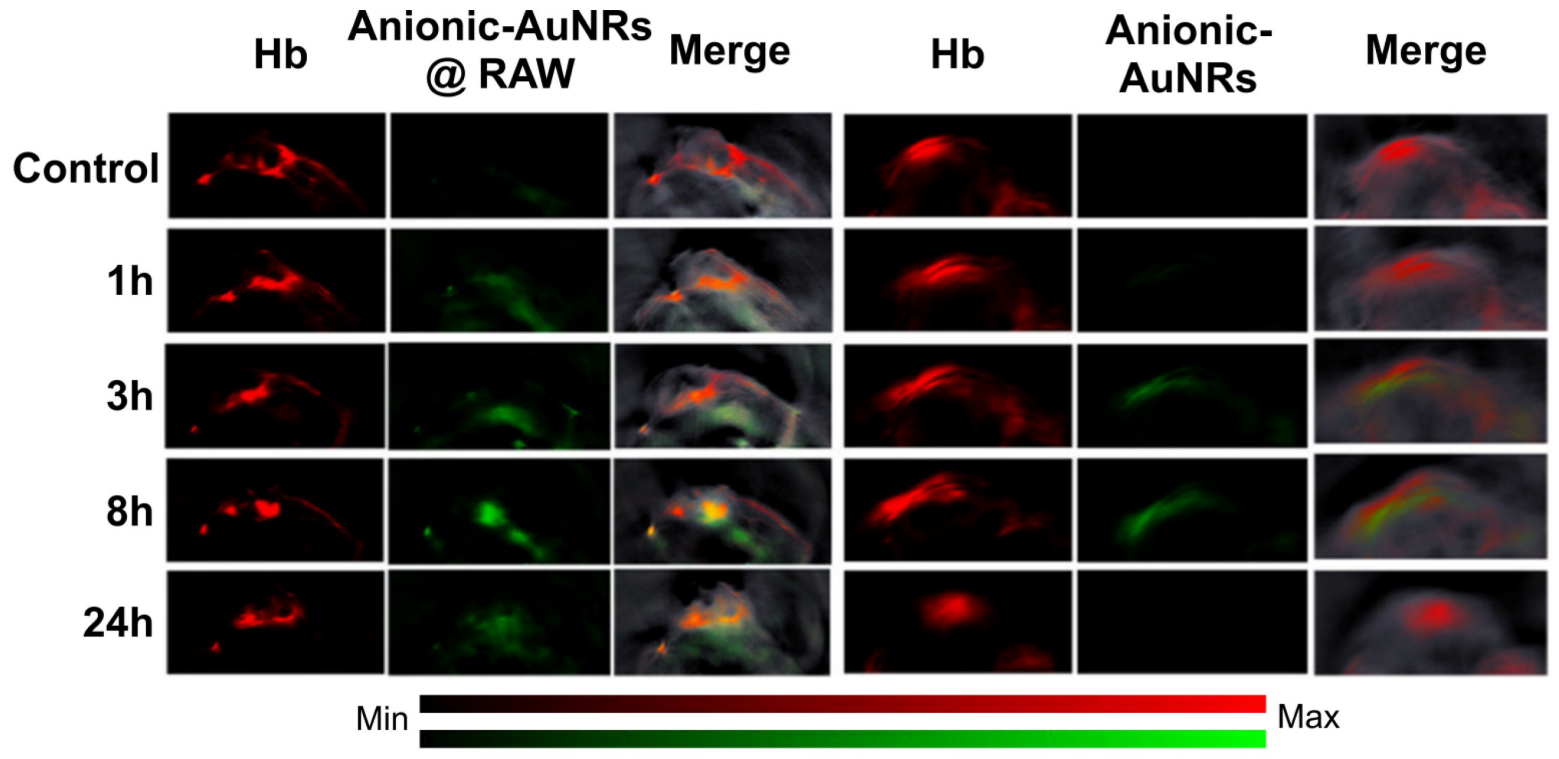
***In-vivo* PAI of tumor bearing mice:** Tumor bearing mice were injected intravenously with Anionic-AuNRs@RAW or Anioninic-AuNRs, over 24 hours. The yellow color is indicative of overlap of Hb and nanoparticles. As can be seen, Anionic-AuNRs@RAW overlapped significantly at 8 and 24 hours, but there was minimal overlap of untargeted Anionic-AuNRs over 24 hours. AuNR: gold nanorod. Hb: deoxygenated hemoglobin. Adapted with permission from 111, copyright 2019, American Chemical Society.

With regards to investigating tumor hypoxia based on molecular level alterations using exogenous contrast agents, gold nanorods are most commonly used as an inorganic contrast agent for PAI 110 as they can be modified to target hypoxia. For example, an *in vivo* study designed macrophage-loaded anionic gold nanoparticles (Anionic-AuNRs@RAW) to image tumor hypoxia in a breast cancer animal model 111. The Anionic-AuNRs incorporated into the macrophages have a plasmon resonance peak at 800 nm with a broader absorbance spectrum than classic gold nanorods (AuNRs). The macrophage-loaded nanoparticles were compared to bare anionic gold particles without target-seeking macrophages (anionic-AuNRs). This contrast agent had maximum accumulation at 8 hours post injection. It was observed that, post-injection, Anionic-AuNR@RAW had a higher relative signal intensity than anionic-AuNR, and the macrophage component allowed the gold nanoparticles to move more efficiently into the tumor and towards the hypoxic region (See Fig. 11). Eventually there was a partial overlap of PA signals between anionic-AuNR@RAW and deoxyhemoglobin (Hb), whereas, this overlap was not seen with anionic-AuNRs. This phenomenon is because anionic-AuNRs@RAW tended to the hypoxic regions of tumors through active macrophage vectors as a “Trojan Horse” to specifically target tumor hypoxic regions, whereas Anionic-AuNRs accumulate at tumor sites through the passive uptake-and-retention effect within the tumor 111.

A similar strategy was pursued by modifying gold nanorods to have 2-nitroimidazole derivatives (NI's), thus making AuNR-NIs 110. The purpose of the NI derivative is to allow for accumulation in hypoxic regions of the tumor. This contrast agent takes advantage of the overexpression of nitroreductases in tumor hypoxia, as AuNR-NI is reduced in hypoxic conditions. This study showed strong PA signals due to the accumulation of these agents whereas, the control mice with just AuNRs showed ambiguous signaling 110. A clever implementation of the overexpression of nitroreductases in tumor hypoxia used Au nanoparticles that aggregated (polymerized) in the presence of the hypoxic environment 102. After aggregation, the plasmon coupling effects between adjacent nanoparticles extended system absorption from the visible into the NIR-II light range. The aggregated particles also enhanced the radiotherapeutic effect.

Liposomes conjugated with inorganic compounds and other nanoparticles have been shown to be effective contrast agents 112. Researchers prepared AQ4N which is a hypoxia responsive drug with liposomes thus creating, AQ4N-hCe6-liposomes. Upon injection in tumor bearing mice, excitation at 680nm increased PAsignals by 2.5 times. This is in comparison to mice with no probe injection, demonstrating this contrast agent accumulates in tumors and can be a tool for *in vivo* tracking 112. Another example of identifying and analyzing hypoxic tumors coupled the photosensitizer chlorin e6 with an anticancer drug (OTS964) in hypoxia-targeting nanoparticles that released the drug within the tumor environment. PA imaging demonstrated that the NPs accumulated within the tumors, and could be used for targeting to facilitate tumor laser irradiation 6. Lipophilic molecules conjugated with magnetic nanomaterials were incorporated into pegylated magnetic ion coordinated nanoplatforms (MICN-PEGs) 113. The metallic ions enable imaging by PA or MRI, and could enhance photothermal therapy. Significantly, the MICN platforms degrade unless in highly acidic environments, such as hypoxic tumors, and even in hypoxic tumors, degrade 24 hours after administration, reducing concerns about long-term toxicity.

## 6. Discussion: Challenges and Future Prospects

According to the published research, PAI is an effective tool to investigate tumor hypoxia. Label free, organic, and inorganic contrast agents are all effective, but each has advantages and disadvantages. A comparison among the contrast agents used in PAI, and corresponding tumor types that have been investigated, are summarized in Table 2.

The differences in methodologies and study setups make direct comparison among the various methods difficult. Therefore, the format for the discussion is to explain the application of PAI in characterizing hypoxia with endogenous and exogenous contrast agents by measuring physiological parameters, followed by the use of contrast enhanced PAI to co-localize with tumors.

Our review has shown that measurement of physiological parameters sO_2_ and MrO_2_ can be achieved through the use of endogenous chromophores, HbO_2_ and Hb. According to the method explained in Section 2, at least a pair of wavelengths (584,590; 750,850; 680, 808; 700-900nm as reported in the reviewed articles) are carefully chosen to illuminate the target area and based on the corresponding absorption coefficients and the concentration of endogenous chromophores, sO_2_ can be quantified. For MrO_2_ and OEF calculations, an additional wavelength (605nm) was also utilized. Contrary to expectations, there does not appear to be a direct correlation between increases or decreases in OEF or sO_2_ and MrO_2_. Importantly, it was found that early-stage cancers may be hyperoxic instead of hypoxic in spite of hypermetabolism, possibly due to local angiogenesis and tumor size 14. Label free imaging allows the visualization of uniquely hypoxic regions. However, it provides limited information on the likelihood of metastases as well as the unique heterogeneity of oxygen distribution within a tumor. More detailed information may be provided by exogenous contrast agents.

pO_2_ and sO_2_ can be evaluated based on unique absorption spectra of exogenous contrast agents. Methylene blue (organic dye) was utilized at 680 and 810nm to evaluate pO_2_. For sO_2_ calculation, usNP-MnO_2_ and GE11-PDA-Pt@USPIOS, which are inorganic contrast agents, were utilized in the range of 750-850nm, whereas HC/PN/DOX-NP was used at 660nm.

**Figure 12:**
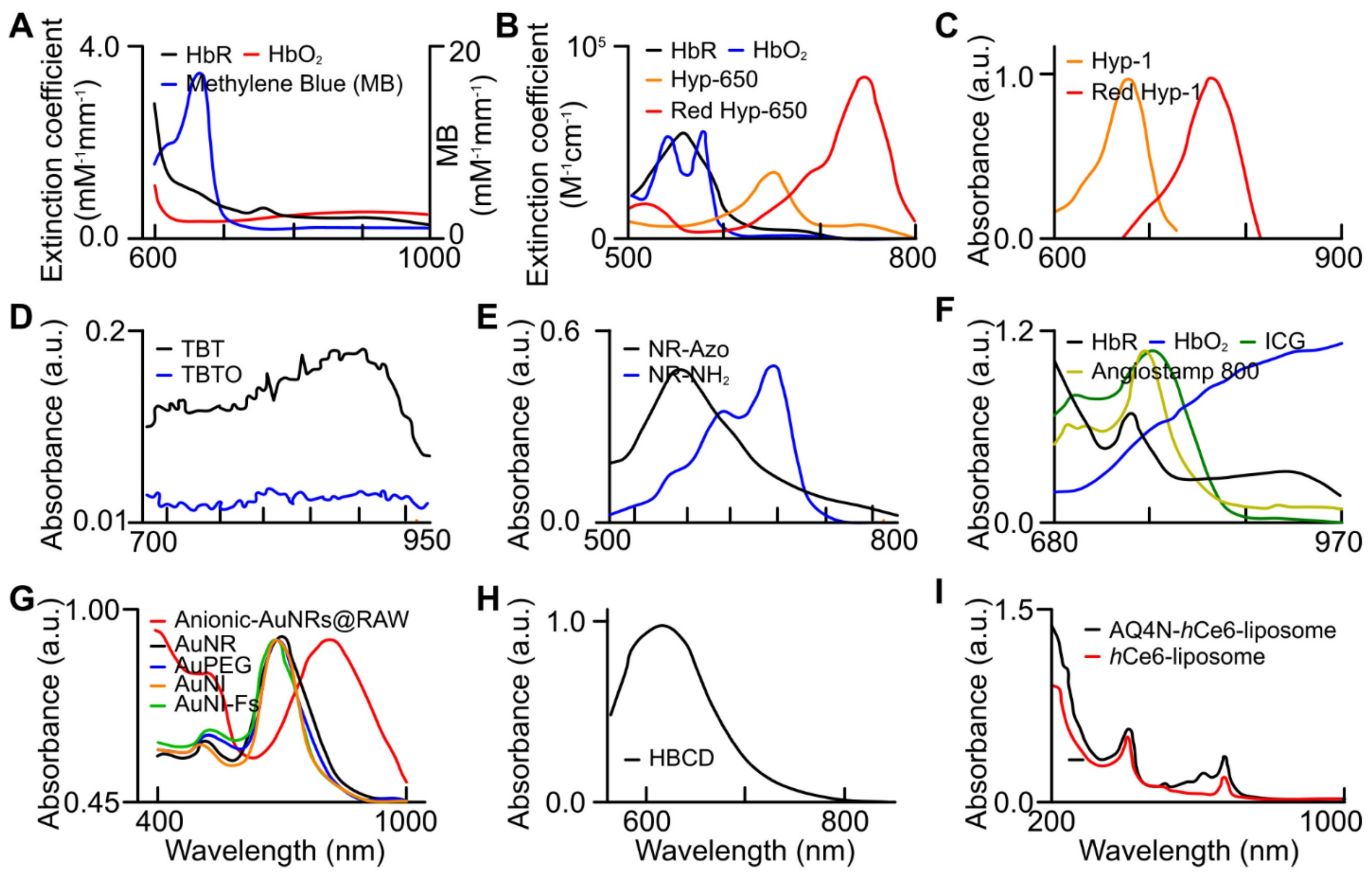
** Absorption spectra of endogenous biomarkers and exogenous (organic and inorganic) contrast agents utilized for tumor hypoxia assessment. (a)** oxy- (red), deoxy-hemoglobin (black), and MB (blue) 99, **(b)** oxy- (blue), deoxy-hemoglobin (black), HyP-650 (green), and Red HyP-650 (red) 105, **(c)** HyP-1 (green), and Red HyP-1 (red) 104, **(d)** TBT (black) and TBTO (blue) 106, **(e)** NR-Azo (black) and NR-NH_2_ (blue) 107, **(f)** oxy- (blue), deoxy-hemoglobin (black), ICG, and Angiostamp 800 88, **(g)** Anionic-AuNRs@RAW (red) 111, AuNRs (black), AuPEG (blue), AuNI (orange), and AuNI-Fs (green) 110, **(h)** HBCD 97, and **(i)** AQ4N-*h*Ce6-liposome (black) and *h*Ce6-liposome (red) 112. AuNI: gold nanoparticles nitroimidazole units, AuNI-Fs: gold nanoparticles nitroimidazole units and fluorescent ATTO-647, AuPeg: gold nanoparticles with polyethylene glycol, AuNR: gold nanorods, HBCD: *hypocrella bambusae* prepared with carbon dots, HbO_2_: oxyhemoglobin, HbR: deoxyhemoglobin, ICG: indocyanine green, TBT: Triphenylamine-benzo thiadiazole diethylamine, TBTO: Triphenylamine-benzo thiadiazole diethylamino oxide. (a) is adapted with permission from 99, copyright 2013, SPIE. (b) is adapted with permission from 105, copyright 2019, Optica. (c) is adapted with permission from 104, copyright 2017, Nature. (d) is adapted with permission from 106, copyright 2021, Cell Press. (e) is adapted from 107, copyright 2019. (f) is adapted from 88, copyright 2020. (g) is adapted with permission from 111 and 110, copyright 2019, Royal Society of Chemistry and American Chemical Society. (h) is adapted with permission from 97, copyright 2018, Elsevier. (i) is adapted with permission from 112, copyright 2017, American Chemical Society.

Exogenous contrast agents that co-localize with specific tumor microenvironment molecules undergo conformational changes that form predictable absorption characteristics. Organic contrast agents, HyP-1 (670 to 760nm) and HyP-650 (650 to 740nm) exhibit unique absorbance peak shifts (fig. 12) when exposed to hypoxic regions and undergo a nitroreductase reaction facilitated by CYP450 proteins 119. CYP450 and its expression is known to be upregulated in tumor hypoxia. The expression of a particular CYP450 is associated with specific types of cancer 120. For example, CYP2S1 is seen to be overexpressed in HepG2 cells exposed to hypoxic conditions. This raises the question - whether dyes can be created which are specific to a certain cancers' CYP expression. Furthermore, upregulation of CYP450s cannot be exclusively attributed to hypoxia. Therefore, there is a need to develop exogenous contrast agents which show a higher degree of specificity for enzymes they target or their mechanism of activation. Under similar conditions to HyP-1, TBTO is transformed into TBT and NR-azo into NR-NH_2_ demonstrating absorption peak shifts from 600 to 740nm and 575 to 710nm respectively. However, other organic agents, ICG based dye and Angiostamp800, is sensitive to overexpressed protein integrin α_v_β_3_ in tumor hypoxia, exhibiting high absorption at ~800nm (see Fig. 12).

Inorganic contrast agents such as gold nanoparticles can be useful in determining hypoxia as they can be modified to be guided towards key hypoxic regions within a tumor. Gold nanoparticles may be more advantageous to use as they can be used for drug delivery and re-oxygenation of hypoxic regions. AuNRs (both anionic and nitro-reductase based) provide exciting strategies to assess hypoxia. Absorption spectra of various gold nanoparticles are shown in Fig. 12.

While exogenous contrast agents can provide PA images with enhanced contrast, there is a variance in efficacy between inorganic and organic contrast agents. In Table 3, properties of exogenous contrast agents used to investigate tumor hypoxia are summarized in terms of their evaluation approach.

Exogenous contrast agents (organic and inorganic) can take advantage of unique features (both physiological and proteomic alterations) of the hypoxic microenvironment to determine the heterogeneity of hypoxia within the tumor itself. Furthermore, exogenous contrast agents can potentially have more robust clinical applications than label free imaging. As the heterogeneity of hypoxia is more visible with these agents, clinicians could potentially predict which lesions will metastasize, as hypoxia promotes a metastatic phenotype 116. While both organic and inorganic exogenous contrast agents can potentially be of use to investigate tumor hypoxia, there is a lack of literature detailing the safety profile findings of using inorganic agents in PAI 38. Even though cyanide-based dyes have been deemed safe by the FDA, there are continued efforts to improve organic contrast agents that use nitroreductases to image tumor hypoxia 38, providing a greater degree of specificity. Therefore, from an imaging perspective, contrast agents that are processed by nitroreductases remain as the better options over nanoparticles. In addition, PAI with organic contrast agents provide a safer option to achieve a better overview of hypoxia status.

The main advantage of PA technology is its potential use in the clinic because it is non-invasive, radiation-free and most importantly, has good penetration depth (recent research has proved several cm imaging depth can be achieved in biological tissue 121). While PA detectability of endogenous markers of hypoxia has proven its usefulness at identifying tumors, it does not demonstrate the same resolution as high-resolution imaging modalities such as fluorescent microscopy. Moreover, in comparison with some established modalities such as MRI, PA imaging is much lower cost, requires much lower maintenance, and almost no training is needed because most clinicians are familiar with ultrasound imaging (which uses the same technology as PA imaging for signal detection).

In addition to PA ability to study hypoxia using endogenous chromophores, its improved detectability using exogenous contrast agents holds the promise for extension to more applications. The ideal contrast agent for PA imaging should be highly absorbent (low quantum yield), highly stable, highly specific, easy to administer, accessible, low cost, and biocompatible for rapid regulatory approval. There is no single contrast agent that meets all the ideal characteristics for every application. The choice of contrast agent for PAI depends on the specific imaging target, imaging system, and application requirements. Further research and development in this field is needed to identify and optimize contrast agents for hypoxia.

One limitation of PAI is the need for a coupling medium, such as water or ultrasound gel. In some applications of PA, safely depositing sufficient light energy into the tissue to the required depth is not straightforward. Nevertheless, PA imaging is becoming more common in research labs and development of FDA-approved hypoxia-targeted PA contrast agents should open up many clinical applications for both tumor imaging and adjuvant theranostic applications.

## 7. Conclusions

Tumor hypoxia is caused by an insufficient blood supply to a growing tumor. Typically, the tumor peripheral region consists of irregular and dense vasculatures as compared to the core. This dysregulated tumor angiogenesis and lack of perfusion towards the tumor core perpetuates hypoxia and triggers the aggressive phenotypes of cancer cells which could suggest that hypoxia is linked to an increased risk of metastases. In addition, several studies have shown that acute hypoxia corresponds to an increased resistance to chemotherapy and radiotherapy. Consequently, this phenomenon contributes to decreased survival rates for cancer patients with severe tumor hypoxia. Therefore, a highly sensitive, yet non-invasive method, such as PAI is required to study the impact of hypoxia in tumor progression and metastasis. Tumor hypoxia has been investigated with PAI technology to assess physiologic and proteomic phenomena. Endogenous contrast agents (i.e., oxy and deoxy hemoglobin - label free), and several organic and inorganic exogenous contrast agents have been explored to map hypoxic regions using PAI. In label-free imaging, hypoxia is primarily studied based on the relation between hemoglobin concentration, oxygenation, and blood perfusion. We now can see that the information provided by label free PAI is enough to determine local sO_2_ and MrO_2_. The advantage of the label-free imaging is that the entire process is non-invasive, however, extraction of hypoxia heterogeneity within the tumor region is not feasible. In addition, we found no studies reporting the correlation between label-free hypoxia and metastasis. Unlike label-free PA imaging, several organic and inorganic agents have been utilized to extract subtle hypoxia changes within the tumor microenvironment and changes to physiological parameters (sO_2_ and pO_2_). Organic agents are less risky due to fast clearing as compared to inorganic counterparts. Moreover, organic contrast agents allow better understanding of dysregulated enzyme concentration in the microenvironment and the impact on the disease state. Lastly, studies involving the use of inorganic contrast agents have showcased the potential clinical applications of PAI - particularly the oxygen distribution gradient within the tumor. Since contrast agent-based PA imaging exhibited great potential towards understanding tumor hypoxia, further exploration will potentially allow a more accurate depiction of tumor progression towards possible metastasis, metastasis staging, and appropriate treatment plans. Overall, current literature has shown PAI to be a promising modality in research with a great potential in clinical applications which can lead to more effective treatment plans, enhanced drug development and delivery, and improved patient outcomes.

## Figures and Tables

**Figure 7:**
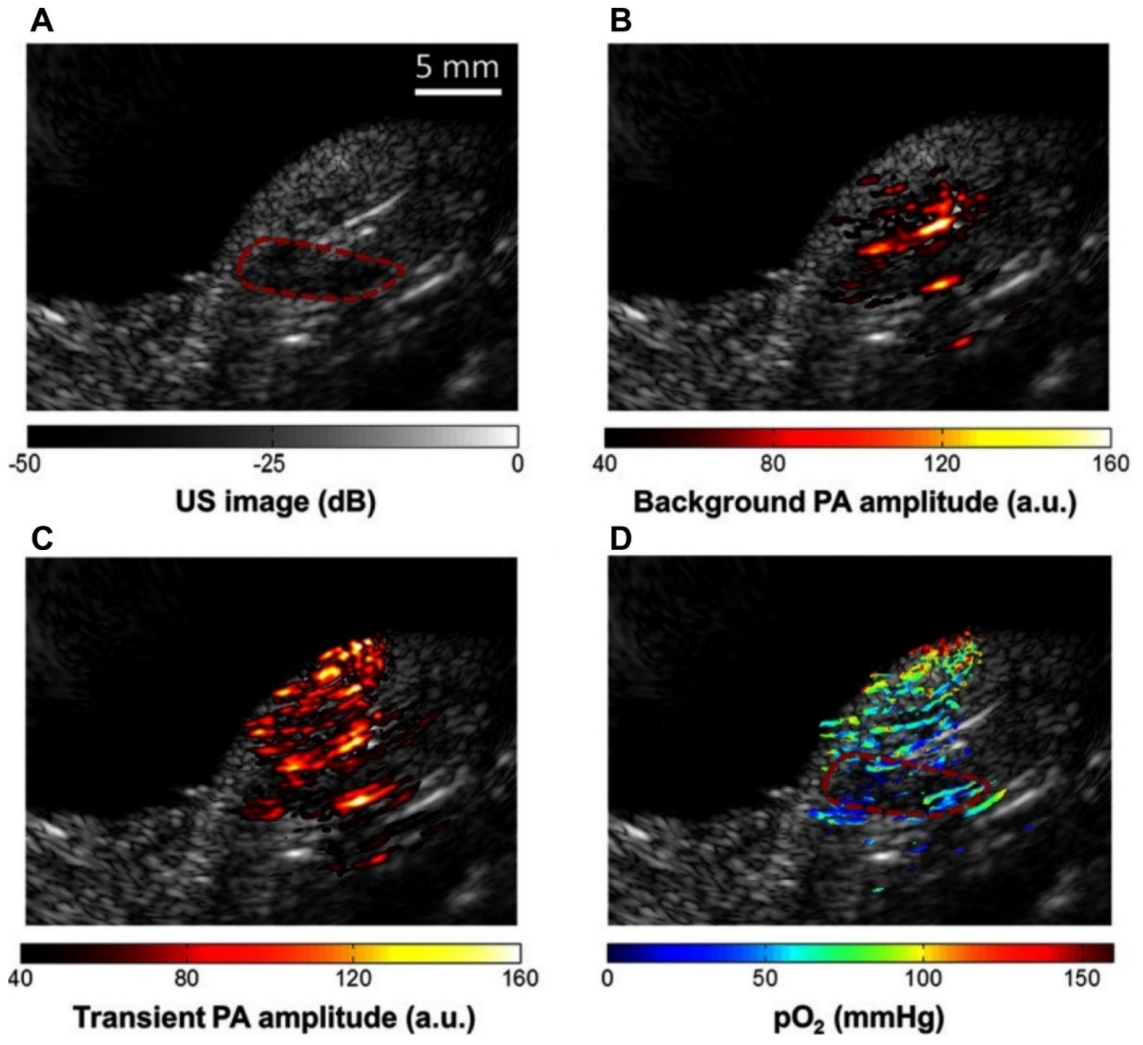
**Methylene blue (MB) contrast agent based two laser method of pO_2_ measurement using PAI (a)** Reference ultrasound image of the left tumor-bearing hindlimb of a mouse. The area of the tumor is enclosed by a red dashed line. **(b)** PAI representing the amplitude of background absorption at 810nm. Amplitude is displayed in linear scale. **(c)** PAI of the transient absorption of MB with an 810-nm laser at a pump-probe delay of 0.25 *μ*s. **(d)** PAI of in color scale superimposed on US image. MB: methylene blue, pO_2_: partial pressure of oxygen, US: ultrasound. Adapted with permission from 99, copyright 2013, SPIE.

**Table 1:** Summary of Imaging Modalities Used to Investigate Tumor Hypoxia

Method		Mechanism of action	Method and component of Hypoxia that is Measured	What information this method provides about tumor hypoxia	Clinical/ Pre-clinical
Phosphorescence quenching		Direct	Local oxygen concentration that is proportional to emission intensity decay	Real-time tissue oxygenation profile	Clinical (in Europe)
^19^F-MRI relaxometry		Direct	Perfluorinated molecular probes to quantify tissue oxygen concentration	Local oxygen tension	Pre-clinical
Overhauser-enhanced MRI		Direct	Saturation of the electron spin of a paramagnetic oxygen-sensitive contrast agent that makes the water protons in tissue via dynamic nuclear polarization to quantify both hypoxia and microvascular permeability	Concentration of the contrast agent and local oxygen concentration	Pre-clinical
MRI variations	DCE-MRI	Indirect (physiologic)	Function of blood perfusion, vascular density, tissue permeability, and extracellular volume fraction, a parameter closely related to cell density	Tissue perfusion	Pre-clinical
	BOLD	Indirect (physiologic)	Deoxyhemoglobin creates a signal enhancement and accelerates spin-spin relaxation time (T2) 9 From which the transverse relaxation rate of water in blood and surrounding tissue can be found from weighted T2 9	This provides a qualitative measurement of hypoxia as a higher relaxation value is indicative of increased perfusion, meaning a greater degree of hypoxia 9	Pre-clinical
Positron emission tomography (PET)		Indirect (physiologic)	Normoxia allows tracers to be reduced by cytochrome or nitro-reductase and diffuse freely in blood 9 Lack of oxygen in hypoxic conditions causes an accumulation of tracers to localize intracellularly, by de-chelation or covalent attachment to thiol rich proteins 9	The degree of uptake of tracer will be indicative of PO_2_, for example uptake of nitroimidazole-based tracers are indicative of pO_2_ < 10 mmHg 9	Pre-clinical (except Contrast enhanced CT)
Near-infrared spectroscopy (NIRS)		Indirect (physiologic)	Oxygenated and deoxygenated hemoglobin have unique absorption spectra thus being uniquely quantifiable 9	Allows to determine the ratio of Hb/HbO_2_ from which a saturation curve can be derived, providing pO_2_ 9	Clinical
*In vivo* bioluminescence imaging		Indirect (endogenous)	HIF-1α is a gene that is upregulated in hypoxia 40 Creating a reporter construct which includes this gene and interacts with luciferase can be used to measure HIF-1α 40	Light emission in animal tumor models shows the degree of expression of HIF-1α which provides a qualitative measurement of hypoxia 40	Research
*In-vivo* fluorescent microscopy		Indirect (physiologic)	Many fluorophores have been developed. For example, fluorophores coupled to nitroreductase (NTR) for 2-photon microscopy or coupled to NIR fluorophores for deeper penetration	Probes show high selectivity and sensitivity but poor light penetration depth	Research
PIMO, EF5		Indirect (physiologic)	Exogenous marker for hypoxia detected by flow cytometry or immunohistochemical techniques	Relation between PIMO accumulation and oxygen tension	Research
Photoacoustic imaging (PAI)		Indirect (physiologic, endogenous)	Oxygenated and deoxygenated hemoglobin have unique absorption spectra thus being uniquely quantifiable, overexpression of exogenous contrast when bound to oxygen sensitive proteins	Oxygenated and deoxygenated hemoglobin, sO_2_, pO_2_, MrO_2_	Pre-clinical

**Table 2:** Comparison of contrast agents used in PAI to investigate tumor hypoxia

Contrast Agent	Benefits	Limitations	Tumor(s) Investigated
Endogenous markers	Non-invasive 38 Able to determine hypoxic regions 79, 83, 84, 86-88 no risk associated to patient unlike exogenous agents 38	cannot show variability within hypoxic regions 79 cannot predict metastasis 79	Melanoma 14 Colon carcinoma 114 Hepatocarcinoma 88, 114 Breast carcinoma 115, 116, 105 Mantle cell lymphoma 86 Glioblastoma 14 Lymphoma 117 Pancreatic carcinoma 89
Exogenous - Organic Contrast Agents	cyanide based dyes are FDA approved 38 fast tissue clearance (safe for patients) 38 106 can cross BBB 38 shows heterogeneity of oxygen in hypoxic regions 6, 106, 107, 108, 118 high biocompatibility 38 carbon based nanomaterials have high photothermal stability 97	quick clearance time results in short imaging window 38 low photothermal stability 38	Breast carcinoma 104 119 Cervical carcinoma 91 Colon carcinoma 88 Hepatocarcinoma 88 107
Exogenous - Inorganic Contrast Agents	slow tissue clearance allowing long imaging window 38 can be used for drug delivery 9 high biocompatibility 38	no inorganic agents are FDA approved 38 slow tissue clearance can cause harm to patients 38 poor photothermal stability (metallic based agents) 38	Colon carcinoma 110 Breast cancer 115,111 Head and neck squamous carcinoma 100

**Table 3:** Properties of organic and inorganic contrast agents used to investigate tumor hypoxia.

Approach	Contrast Agent		Type		Mechanism of Activation	Absorbance
Physiological hemodynamic change	Methylene blue		Organic		Oxygen sensitive properties cause it to turn blue in the presence of oxygen 99	Methylene blue = 650nm 99 Oxygen activated methylene blue = 810nm 99
	AuNP-MnO_2_		Inorganic		Metallic nanomaterial 100	React with ROS to make H_2_O_2_ and O_2_ to reoxygenate hypoxic tumor 100
	GE11-PDA-Pt@USPIOS		Inorganic		Metallic nanomaterial 101	Decompose H_2_O_2_ to O_2_, to reoxygenate tumor 101
	HC/PN/DOX-NP		Inorganic		Polymer based nanomaterial 103	Hypoxic condition will cause agent disassemble leading to doxorubicin release for chemotherapy 103
Quantification of molecular transformation or activation	HyP-1		Organic		Reduced by CYP450 104	HyP-1 = 670nm 104 Red-HyP-1 = 760nm 104
	HyP-650		Organic		Reduced by CYP450 105	HyP-650 = 650nm 105 Red-HyP-650 = 740nm 105
	TBTO		Organic		Reduced by CYP450 106	TBTO = 600nm 106 TBT = 760nm 106
	NR-Azo		Organic		Cleavage of Azo moiety by azoreductases 107	NR-Azo = 575nm 107 NR-NH_2_ = 710nm 107
	As-Cy-NO2		Organic		Cleavage of nitrobenzene moiety by nitroreductases 108	As-Cy-NO_2_ = 670nm 108 As-Cy-1 = 730nm 108
	Angiostamp800		Organic		Binds to αvβ_3_ which releases dye 88	Angiostamp800 = 800nm 88
	Anionic-AuNRs@RAW		Inorganic		Metallic Nanomaterial 111	Macrophage component of agent guides towards hypoxic regions of tumor for imaging 111
	AuNR-NI		Inorganic		Metallic Nanomaterial 110	Nitroreductase overexpression will interact with NI, resulting in accumulation of agent in tumor for imaging 110
	HBCD		Organic		Carbon-based nanomaterial 97	HB generates oxygen, to reoxygenate tumor 97
	AQ4N-hCe6-liposomes			Inorganic	Transition metal chalcogenides 112	Hypoxia triggers activation of AQ4N leading to tumor inhibition 112
		OTS964/Ce6@NPs		Organic	Photosensitizer for PA guided photodynamic therapy (PDT) coupled with chemotherapy	NP disintegrates within hypoxic tumor, releasing chemotherapy whose efficacy is enhanced by PA-guided PDT 40
	MICN-PEGs		Inorganic		Magnetic ion platforms (for PA or MRI) degrade unless within hypoxic environment, can enhance PDT.	MICN-PEGs degrade unless within hypoxic tumor, enabling tumor targeting by PA or MRI 113
						

## Abbreviations

PA: Photoacoustic
PAI: Photoacoustic imaging
HIF-1/2: hypoxia inducible transcription factors
EMT: epithelial to mesenchymal transformation
NTR: Nitroreductase
PET: Positron emission tomography
NIRS: Near infrared spectroscopy
MRI: Magnetic resonance imaging
EMT: Epithelial to mesenchymal transformation
NTR: Nitroreductase
sO_2_: Oxygen saturation concentration
pO_2_: partial pressure of oxygen
MrO_2_: metabolic rate of oxygen
PIMO: pimonidazole
CBP: creb binding protein
HRE: hypoxia-responsive element
VEGF: vascular endothelial growth factor
GLUT-1: glucose transporter 1
NF-kB: nuclear factor kappa-light-chain-enhancer of activated B cells
STAT3, p53: signal transducer and activator of transcription 3
EPR: electron paramagnetic resonance
MRS: magnetic resonance spectroscopy
OMRI: Overhauser-enhanced MRI
DCE-MRI: dynamic contrast enhanced-MRI
BOLD-MRI: blood oxygenation level-dependent MRI
PET: positron emission tomography
NIRS: Near Infrared Spectroscopy
Hb: hemoglobin
HbO_2_: oxyhemoglobin ()
IHC): immunohistochemical
NTR: nitroreductase
fMRI: functional magnetic resonance imaging
OEF: oxygen extraction factor
NIR: near-infrared
PDT: photodynamic therapy
MSOT: multi-spectral optoacoustic tomography
HBCDs: hypocrella bambusae carbon dots
TMC: transition metal chalcogenides
MB: methylene blue
ROS: reactive oxygen species
CYP450: cytochromes P450
TBTO: Triphenylamine-benzo thiadiazole diethylamino oxide
TBT: triphenylamine-benzo thiadiazole diethylamine
AuNR-Nis: Gold nanorod n2-nitroimidazole
AuNRs: Gold nanorods
MICN-PEGs: pegylated magnetic ion coordinated nanoplatforms
BBB: blood brain barrier
AuPeg: Gold nanoparticles with polyethylene glycol

## References

[1] Lin W, Wu S, Chen X, Ye Y, Weng Y, Pan Y (2020). Characterization of Hypoxia Signature to Evaluate the Tumor Immune Microenvironment and Predict Prognosis in Glioma Groups. Front Oncol.

[2] Jain RK (2005). Normalization of tumor vasculature: an emerging concept in antiangiogenic therapy. Science.

[3] Muz B, de la Puente P, Azab F, Azab AK (2015). The role of hypoxia in cancer progression, angiogenesis, metastasis, and resistance to therapy. Hypoxia (Auckl).

[4] Jing X, Yang F, Shao C, Wei K, Xie M, Shen H (2019). Role of hypoxia in cancer therapy by regulating the tumor microenvironment. Mol Cancer.

[5] Chang WH, Forde D, Lai AG (2019). A novel signature derived from immunoregulatory and hypoxia genes predicts prognosis in liver and five other cancers. J Transl Med.

[6] Shi G, Cui Y, Zhao J, Liu J, Wang Y, Yang Y (2023). Identifying TOPK and Hypoxia Hallmarks in Esophageal Tumors for Photodynamic/Chemo/Immunotherapy and Liver Metastasis Inhibition with Nanocarriers. ACS Nano.

[7] Munn LL, Jain RK (2019). Vascular regulation of antitumor immunity. Science.

[8] Nobre AR, Entenberg D, Wang Y, Condeelis J, Aguirre-Ghiso JA (2018). The Different Routes to Metastasis via Hypoxia-Regulated Programs. Trends Cell Biol.

[9] Walsh JC, Lebedev A, Aten E, Madsen K, Marciano L, Kolb HC (2014). The clinical importance of assessing tumor hypoxia: relationship of tumor hypoxia to prognosis and therapeutic opportunities. Antioxid Redox Signal.

[10] Liu J, Liu Z, Wu D (2019). Multifunctional hypoxia imaging nanoparticles: multifunctional tumor imaging and related guided tumor therapy. Int J Nanomedicine.

[11] McKeown SR (2014). Defining normoxia, physoxia and hypoxia in tumours-implications for treatment response. Br J Radiol.

[12] Hunter FW, Wouters BG, Wilson WR (2016). Hypoxia-activated prodrugs: paths forward in the era of personalised medicine. Br J Cancer.

[13] Vordermark D, Brown JM (2003). Endogenous markers of tumor hypoxia predictors of clinical radiation resistance?. Strahlenther Onkol.

[14] Yao J, Maslov KI, Zhang Y, Xia Y, Wang LV (2011). Label-free oxygen-metabolic photoacoustic microscopy in vivo. J Biom Opt.

[15] Lee C-T, Boss M-K, Dewhirst MW (2014). Imaging tumor hypoxia to advance radiation oncology. Antioxid Redox Signal.

[16] Egeland TA, Gulliksrud K, Gaustad JV, Mathiesen B, Rofstad EK (2012). Dynamic contrast-enhanced-MRI of tumor hypoxia. Magn Reson Med.

[17] Cai X, Zhu Q, Zeng Y, Zeng Q, Chen X, Zhan Y (2019). Manganese Oxide Nanoparticles As MRI Contrast Agents In Tumor Multimodal Imaging And Therapy. Int J Nanomedicine.

[18] Song R, Zhang M, Liu Y, Cui Z, Zhang H, Tang Z (2018). A multifunctional nanotheranostic for the intelligent MRI diagnosis and synergistic treatment of hypoxic tumor. Biomaterials.

[19] Lin L-S, Song J, Song L, Ke K, Liu Y, Zhou Z (2018). Simultaneous Fenton-like Ion Delivery and Glutathione Depletion by MnO2-Based Nanoagent to Enhance Chemodynamic Therapy. Angew Chemie Int Ed Engl.

[20] Dong Z, Liang P, Guan G, Yin B, Wang Y, Yue R (2022). Overcoming Hypoxia-Induced Ferroptosis Resistance via a 19F/1H-MRI Traceable Core-Shell Nanostructure. Angew Chemie Int Ed Engl.

[21] Liu J, Cabral H, Song B, Aoki I, Chen Z, Nishiyama N (2021). Nanoprobe-based magnetic resonance imaging of hypoxia predicts responses to radiotherapy, immunotherapy, and sensitizing treatments in pancreatic tumors. ACS Nano.

[22] Fu LH, Wan Y, Li C, Qi C, He T, Yang C (2021). Biodegradable Calcium Phosphate Nanotheranostics with Tumor-Specific Activatable Cascade Catalytic Reactions-Augmented Photodynamic Therapy. Adv Funct Mat.

[23] Mi P, Kokuryo D, Cabral H, Wu H, Terada Y, Saga T (2016). A pH-activatable nanoparticle with signal-amplification capabilities for non-invasive imaging of tumour malignancy. Nat Nanotechnol.

[24] Barjesteh T, Mansur S, Bao Y (2021). Inorganic Nanoparticle-Loaded Exosomes for Biomedical Applications. Molecules.

[25] Lee J-R, Park B-W, Kim J, Choo YW, Kim HY, Yoon J-K (2020). Nanovesicles derived from iron oxide nanoparticles-;incorporated mesenchymal stem cells for cardiac repair. Sci Adv.

[26] Jung KO, Jo H, Yu JH, Gambhir SS, Pratx G (2018). Development and MPI tracking of novel hypoxia-targeted theranostic exosomes. Biomaterials.

[27] Sun X, Niu G, Chan N, Shen B, Chen X (2011). Tumor hypoxia imaging. Mol Imaging Biol.

[28] O'Connor JPB, Robinson SP, Waterton JC (2019). Imaging tumour hypoxia with oxygen-enhanced MRI and BOLD MRI. The Br J Radiol.

[29] Badr CE, Tannous BA (2011). Bioluminescence imaging: progress and applications. Trends Biotechnol.

[30] Lehmann S, Stiehl DP, Honer M, Dominietto M, Keist R, Kotevic I (2009). Longitudinal and multimodal in vivo imaging of tumor hypoxia and its downstream molecular events. Proc Natl Acad Sci U S A.

[31] Feng P, Zhang H, Deng Q, Liu W, Yang L, Li G (2016). Real-Time Bioluminescence Imaging of Nitroreductase in Mouse Model. Anal Chem.

[32] Khalil AA, Jameson MJ, Broaddus WC, Lin PS, Dever SM, Golding SE (2013). The Influence of Hypoxia and pH on Bioluminescence Imaging of Luciferase-Transfected Tumor Cells and Xenografts. Int J Mol Imaging.

[33] Caceres G, Zankina R, Zhu X, Jiao J-a, Wong H, Aller A (2003). Determination of chemotherapeutic activity in vivo by luminescent imaging of luciferase-transfected human tumors. Anticancer Drugs.

[34] Jenkins DE, Oei Y, Hornig YS, Yu SF, Dusich J, Purchio T (2003). Bioluminescent imaging (BLI) to improve and refine traditional murine models of tumor growth and metastasis. Clin Exp Metastasis.

[35] Scatena CD, Hepner MA, Oei YA, Dusich JM, Yu S-F, Purchio T (2004). Imaging of bioluminescent LNCaP-luc-M6 Tumors: A new animal model for the study of metastatic human prostate cancer. Prostate.

[36] Zeamari S, Rumping G, Floot B, Lyons S, Stewart FA (2004). In vivo bioluminescence imaging of locally disseminated colon carcinoma in rats. Br J Cancer.

[37] Zhang C, Yan Z, Arango ME, Painter CL, Anderes K (2009). Advancing bioluminescence imaging technology for the evaluation of anticancer agents in the MDA-MB-435-HAL-Luc mammary fat pad and subrenal capsule tumor models. Clin Cancer Res.

[38] Wang S, Lin J, Wang T, Chen X, Huang P (2016). Recent Advances in Photoacoustic Imaging for Deep-Tissue Biomedical Applications. Theranostics.

[39] Karan S, Cho MY, Lee H, Lee H, Park HS, Sundararajan M (2021). Near-Infrared Fluorescent Probe Activated by Nitroreductase for In Vitro and In Vivo Hypoxic Tumor Detection. J Med Chem.

[40] Saha D, Dunn H, Zhou H, Harada H, Hiraoka M, Mason RP (2011). In vivo bioluminescence imaging of tumor hypoxia dynamics of breast cancer brain metastasis in a mouse model. J Vis Exp.

[41] Manwar R, Li X, Mahmoodkalayeh S, Asano E, Zhu D, Avanaki K (2020). Deep learning protocol for improved photoacoustic brain imaging. J Biophotonics.

[42] Matchynski JI, Manwar R, Kratkiewicz KJ, Madangopal R, Lennon VA, Makki KM (2021). Direct measurement of neuronal ensemble activity using photoacoustic imaging in the stimulated Fos-LacZ transgenic rat brain: A proof-of-principle study. Photoacoustics.

[43] Nasiriavanaki M, Xia J, Wan H, Bauer AQ, Culver JP, Wang LV (2014). High-resolution photoacoustic tomography of resting-state functional connectivity in the mouse brain. Proc Natl Acad Sci U S A.

[44] Zafar M, Manwar R, Avanaki KJJoB (2022). High-fidelity compression for high-throughput photoacoustic microscopy systems. J Biophotonics.

[45] Manwar R, Kratkiewicz K, Avanaki K (2020). Investigation of the effect of the skull in transcranial photoacoustic imaging: a preliminary ex vivo study. Sensors.

[46] Mohammadi-Nejad A-R, Mahmoudzadeh M, Hassanpour MS, Wallois F, Muzik O, Papadelis C (2018). Neonatal brain resting-state functional connectivity imaging modalities. Photoacoustics.

[47] Mahmoodkalayeh S, Jooya HZ, Hariri A, Zhou Y, Xu Q, Ansari MA (2018). Low Temperature-Mediated Enhancement of Photoacoustic Imaging Depth. Sci Rep.

[48] Meimani N, Abani N, Gelovani J, Avanaki MR (2017). A numerical analysis of a semi-dry coupling configuration in photoacoustic computed tomography for infant brain imaging. Photoacoustics.

[49] Manwar R, Hosseinzadeh M, Hariri A, Kratkiewicz K, Noei S, N (2018). Avanaki MR. Photoacoustic Signal Enhancement: Towards Utilization of Low Energy Laser Diodes in Real-Time Photoacoustic Imaging. Sensors.

[50] Manwar R, Lara JB, Prakash R, Ranjbaran SM, Avanaki K (2022). Randomized multi-angle illumination for improved linear array photoacoustic computed tomography in brain. J Biophotonics.

[51] Kratkiewicz K, Manwar R, Zhou Y, Mozaffarzadeh M, Avanaki K (2021). Technical considerations in the Verasonics research ultrasound platform for developing a photoacoustic imaging system. Biomed Opt Express.

[52] Manwar R, McGuire LS, Islam MT, Shoo A, Charbel FT, Pillers D-AM (2022). Transfontanelle photoacoustic imaging for in-vivo cerebral oxygenation measurement. Sci Rep.

[53] Avanaki K, Gelovani JG (2020). Ultrasound and multispectral photoacoustic systems and methods for brain and spinal cord imaging through acoustic windows. Google Patents.

[54] Mahmoodkalayeh S, Kratkiewicz K, Manwar R, Shahbazi M, Ansari MA, Natarajan G (2021). Wavelength and pulse energy optimization for detecting hypoxia in photoacoustic imaging of the neonatal brain: a simulation study. Biomed Opt Express.

[55] Zeng L, Ma G, Lin J, Huang PJS (2018). Photoacoustic probes for molecular detection: recent advances and perspectives. Small.

[56] Ma Y, Xu L, Yin B, Shang J, Chen F, Xu J (2021). Ratiometric semiconducting polymer nanoparticle for reliable photoacoustic imaging of pneumonia-induced vulnerable atherosclerotic plaque in vivo. Nano Lett.

[57] Chen F, Teng L, Lu C, Zhang C, Rong Q, Zhao Y (2020). Activatable Magnetic/Photoacoustic Nanoplatform for Redox-Unlocked Deep-Tissue Molecular Imaging In Vivo via Prussian Blue Nanoprobe. Anal Chem.

[58] Sun L, Ouyang J, Ma Y, Zeng Z, Zeng C, Zeng F (2021). An Activatable Probe with Aggregation-Induced Emission for Detecting and Imaging Herbal Medicine Induced Liver Injury with Optoacoustic Imaging and NIR-II Fluorescence Imaging. Adv Healthc Mater.

[59] Garcia-Uribe A, Erpelding TN, Ke H, Reddy KN, Sharma A, Wang LV (2023). Noninvasive in vivo photoacoustic measurement of internal jugular venous oxygenation in humans. arXiv preprint arXiv:10775.

[60] Ivankovic I, Deán-Ben XL, Lin H-CA, Zhang Z, Trautz B, Petry A (2019). Volumetric optoacoustic tomography enables non-invasive in vivo characterization of impaired heart function in hypoxic conditions. Sci Rep.

[61] Bendinger AL, Glowa C, Peter J, Karger CP (2018). Photoacoustic imaging to assess pixel-based sO2 distributions in experimental prostate tumors. J Biomed Opt.

[62] Brown E, Brunker J, Bohndiek SE (2019). Photoacoustic imaging as a tool to probe the tumour microenvironment. Dis Model Mech.

[63] Cao F, Qiu Z, Li H, Lai P (2017). Photoacoustic imaging in oxygen detection. Appl Sci.

[64] Mehrmohammadi M, Yoon SJ, Yeager D, Emelianov SY (2013). Photoacoustic Imaging for Cancer Detection and Staging. Curr Mol Imaging.

[65] Xu G, Xue Y, Özkurt ZG, Slimani N, Hu Z, Wang X (2017). Photoacoustic imaging features of intraocular tumors: Retinoblastoma and uveal melanoma. PLoS One.

[66] Xu M, Wang LV (2006). Photoacoustic imaging in biomedicine. Rev Sci Instrum.

[67] Yan Y, Gomez-Lopez N, Basij M, Shahvari AV, Vadillo-Ortega F, Hernandez-Andrade E (2019). Photoacoustic imaging of the uterine cervix to assess collagen and water content changes in murine pregnancy. Biomed Opt Express.

[68] Zhang HF, Maslov K, Stoica G, Wang LV (2006). Functional photoacoustic microscopy for high-resolution and noninvasive in vivo imaging. Nat biotechnol.

[69] Siphanto R, Thumma K, Kolkman R, Van Leeuwen T, De Mul F, Van Neck J (2005). Serial noninvasive photoacoustic imaging of neovascularization in tumor angiogenesis. Opt Express.

[70] Mallidi S, Luke GP, Emelianov S (2011). Photoacoustic imaging in cancer detection, diagnosis, and treatment guidance. Trends Biotechnol.

[71] Li M-L, Oh J-T, Xie X, Ku G, Wang W, Li C (2008). Simultaneous molecular and hypoxia imaging of brain tumors in vivo using spectroscopic photoacoustic tomography. Proc IEEE.

[72] Laufer J, Elwell C, Delpy D, Beard P (2005). In vitro measurements of absolute blood oxygen saturation using pulsed near-infrared photoacoustic spectroscopy: accuracy and resolution. Phys Med Biol.

[73] Cox BT, Laufer JG, Beard PC, Arridge SR (2012). Quantitative spectroscopic photoacoustic imaging: a review. J Biomed Opt.

[74] Zafar M, Kratkiewicz K, Manwar R, Avanaki M (2019). Development of low-cost fast photoacoustic computed tomography: System characterization and phantom study. Appl Sci.

[75] Kratkiewicz K, Manwar R, Rajabi-Estarabadi A, Fakhoury J, Meiliute J, Daveluy S (2019). Photoacoustic/ultrasound/optical coherence tomography evaluation of melanoma lesion and healthy skin in a swine model. Sensors (Basel).

[76] Hariri A, Fatima A, Mohammadian N, Mahmoodkalayeh S, Ansari MA, Bely N (2017). Development of low-cost photoacoustic imaging systems using very low-energy pulsed laser diodes. J. Biomed. Opt.

[77] Fatima A, Kratkiewicz K, Manwar R, Zafar M, Zhang R, Huang B Review of Cost Reduction Methods in Photoacoustic Computed Tomography. Photoacoustics. 2019: 100137.

[78] Hariri A, Fatima A, Mohammadian N, Bely N, Nasiriavanaki M (2016). Towards low cost photoacoustic microscopy system for evaluation of skin health. Proc. SPIE 9976, Imaging Spectrometry XXI.

[79] Qin Q, Grgac K, van Zijl PCM (2011). Determination of whole-brain oxygen extraction fractions by fast measurement of blood T(2) in the jugular vein. Magn Reson Med.

[80] Weber J, Beard PC, Bohndiek SE (2016). Contrast agents for molecular photoacoustic imaging. Nat methods.

[81] Smith BR, Gambhir SS (2017). Nanomaterials for In Vivo Imaging. Chem Rev.

[82] Kim C, Erpelding TN, Jankovic L, Pashley MD, Wang LV (2010). Deeply penetrating in vivo photoacoustic imaging using a clinical ultrasound array system. Biomed Opt Express.

[83] Beard P (2011). Biomedical photoacoustic imaging. Interface Focus.

[84] Attia ABE, Balasundaram G, Moothanchery M, Dinish US, Bi R, Ntziachristos V (2019). A review of clinical photoacoustic imaging: Current and future trends. Photoacoustics.

[85] Karmacharya MB, Sultan LR, Kirkham BM, Brice AK, Wood AKW, Sehgal CM (2020). Photoacoustic Imaging for Assessing Tissue Oxygenation Changes in Rat Hepatic Fibrosis. Diagnostics (Basel).

[86] Keša P, Pokorná E, Grajciarová M, Tonar Z, Vočková P, Trochet P (2021). Quantitative In VivoMonitoring of Hypoxia and Vascularization of Patient-Derived Murine Xenografts of Mantle Cell Lymphoma Using Photoacoustic and Ultrasound Imaging. Ultrasound Med Biol.

[87] Choi SS, Mandelis A, Guo X, Lashkari B, Kellnberger S, Ntziachristos V (2016). Wavelength-Modulated Differential Photoacoustic Spectroscopy (WM-DPAS) for noninvasive early cancer detection and tissue hypoxia monitoring. J Biophotonics.

[88] Lavaud J, Henry M, Gayet P, Fertin A, Vollaire J, Usson Y (2020). Noninvasive monitoring of liver metastasis development via combined multispectral photoacoustic imaging and fluorescence diffuse optical tomography. Int J Biol Sci.

[89] Wang Y, Jhang D-F, Tsai C-H, Chiang N-J, Tsao C-H, Chuang C-C (2021). In Vivo Assessment of Hypoxia Levels in Pancreatic Tumors Using a Dual-Modality Ultrasound/Photoacoustic Imaging System. Micromachines.

[90] Hacker L, Else TR, Lefebvre TL, Sweeney PW, Brown EL, Bohndiek SE Photoacoustic imaging reveals differences in perfusion-limited hypoxia in murine breast cancer models (Conference Presentation). Proc. SPIE PC12379, Photons Plus Ultrasound: Imaging and Sensing. 2023: PC123791E.

[91] Basij M, Karpiouk A, Winer I, Emelianov S, Mehrmohammadi M (2021). Dual-Illumination Ultrasound/ Photoacoustic System for Cervical Cancer imaging. IEEE Photonics J.

[92] Mallidi S, Watanabe K, Timerman D, Schoenfeld D, Hasan T (2015). Prediction of tumor recurrence and therapy monitoring using ultrasound-guided photoacoustic imaging. Theranostics.

[93] Neuschmelting V, Kim K, Malekzadeh-Najafabadi J, Jebiwott S, Prakash J, Scherz A (2018). WST11 vascular targeted photodynamic therapy effect monitoring by multispectral optoacoustic tomography (MSOT) in mice. Theranostics.

[94] He G, Li Y, Younis MR, Fu L-H, He T, Lei S (2022). Synthetic biology-instructed transdermal microneedle patch for traceable photodynamic therapy. Nat Commun.

[95] Lei S, Zhang J, Blum NT, Li M, Zhang D-Y, Yin W (2022). In vivo three-dimensional multispectral photoacoustic imaging of dual enzyme-driven cyclic cascade reaction for tumor catalytic therapy. Nat Commun.

[96] Tsang VTC, Li X, Wong TTW (2020). A Review of Endogenous and Exogenous Contrast Agents Used in Photoacoustic Tomography with Different Sensing Configurations. Sensors.

[97] Jia Q, Zheng X, Ge J, Liu W, Ren H, Chen S (2018). Synthesis of carbon dots from Hypocrella bambusae for bimodel fluorescence/photoacoustic imaging-guided synergistic photodynamic/photothermal therapy of cancer. J Colloid Interface Sci.

[98] Cheng MHY, Mo Y, Zheng G (2021). Nano versus Molecular: Optical Imaging Approaches to Detect and Monitor Tumor Hypoxia. Adv Healthc Mater.

[99] Shao Q, Morgounova E, Jiang C, Choi J-H, Bischof J, Ashkenazi S (2013). In vivo photoacoustic lifetime imaging of tumor hypoxia in small animals. J Biomed Opt.

[100] Rich LJ, Damasco JA, Bulmahn JC, Kutscher HL, Prasad PN, Seshadri M (2020). Photoacoustic and Magnetic Resonance Imaging of Hybrid Manganese Dioxide-Coated Ultra-Small NaGdF4 Nanoparticles for Spatiotemporal Modulation of Hypoxia in Head and Neck Cancer. Cancers.

[101] Yang C, Mi X, Su H, Yang J, Gu Y, Zhang L (2019). GE11-PDA-Pt@USPIOs nano-formulation for relief of tumor hypoxia and MRI/PAI-guided tumor radio-chemotherapy. Biomat Sci.

[102] Geng H, Chen K, Cao L, Liu L, Huang Y, Liu J (2023). Hypoxia-Responsive Aggregation of Gold Nanoparticles for Near-Infrared-II Photoacoustic Imaging-Guided Enhanced Radiotherapy. Langmuir.

[103] He H, Zhu R-y, Sun W, Cai K, Chen Y, Yin L (2018). Selective cancer treatment via photodynamic sensitization of hypoxia-responsive drug delivery. Nanoscale.

[104] Knox HJ, Hedhli J, Kim TW, Khalili K, Dobrucki LW, Chan J (2017). A bioreducible N-oxide-based probe for photoacoustic imaging of hypoxia. Nat Commun.

[105] Chen M, Knox HJ, Tang Y, Liu W, Nie L, Chan J (2019). Simultaneous photoacoustic imaging of intravascular and tissue oxygenation. Opt Lett.

[106] Li M, Li H, Wu Q, Niu N, Huang J, Zhang L (2021). Hypoxia-activated probe for NIR fluorescence and photoacoustic dual-mode tumor imaging. iScience.

[107] Huang J, Wu Y, Zeng F, Wu S (2019). An Activatable Near-Infrared Chromophore for Multispectral Optoacoustic Imaging of Tumor Hypoxia and for Tumor Inhibition. Theranostics.

[108] Zhang S, Chen H, Wang L, Qin X, Jiang B-P, Ji S-C (2022). A General Approach to Design Dual Ratiometric Fluorescent and Photoacoustic Probes for Quantitatively Visualizing Tumor Hypoxia Levels In Vivo. Angew Chemie Int Ed Engl.

[109] Pan D, Pramanik M, Senpan A, Allen JS, Zhang H, Wickline SA (2011). Molecular photoacoustic imaging of angiogenesis with integrin-targeted gold nanobeacons. FASEB J.

[110] Umehara Y, Kageyama T, Son A, Kimura Y, Kondo T, Tanabe K (2019). Biological reduction of nitroimidazole-functionalized gold nanorods for photoacoustic imaging of tumor hypoxia. RSC Adv.

[111] An L, Wang Y, Lin J, Tian Q, Xie Y, Hu J (2019). Macrophages-Mediated Delivery of Small Gold Nanorods for Tumor Hypoxia Photoacoustic Imaging and Enhanced Photothermal Therapy. ACS Appl Mater Interfaces.

[112] Feng L, Cheng L, Dong Z, Tao D, Barnhart TE, Cai W (2017). Theranostic Liposomes with Hypoxia-Activated Prodrug to Effectively Destruct Hypoxic Tumors Post-Photodynamic Therapy. ACS Nano.

[113] Yang Y, Yang T, Chen F, Zhang C, Yin B, Yin X (2022). Degradable magnetic nanoplatform with hydroxide ions triggered photoacoustic, MR imaging, and photothermal conversion for precise cancer theranostic. Nano Lett.

[114] Baik JW, Kim H, Son M, Choi J, Kim KG, Baek JH (2021). Intraoperative label-free photoacoustic histopathology of clinical specimens. Laser Photon Rev.

[115] Ron A, Deán-Ben XL, Gottschalk S, Razansky D (2019). Volumetric Optoacoustic Imaging Unveils High-Resolution Patterns of Acute and Cyclic Hypoxia in a Murine Model of Breast CancerImaging High-Resolution Patterns of Acute and Cyclic Hypoxia. Cancer Res.

[116] Gilkes DM (2016). Implications of Hypoxia in Breast Cancer Metastasis to Bone. Int J Mol Sci.

[117] Placke J-M, Mertens D, Tasdogan A, Chorti E, Schadendorf D, Ugurel S (2023). Multispectral optoacoustic tomography to differentiate between lymph node metastases and coronavirus-19 vaccine-associated lymphadenopathy. J Eur Acad Dermatol Venereol.

[118] Zheng X, Mao H, Huo D, Wu W, Liu B, Jiang X (2017). Successively activatable ultrasensitive probe for imaging tumour acidity and hypoxia. Nat Biomed Eng.

[119] Fan L, Zan Q, Lin B, Wang X, Gong X, Zhao Z (2020). Hypoxia imaging in living cells, tissues and zebrafish with a nitroreductase-specific fluorescent probe. Analyst.

[120] Elfaki I, Mir R, Almutairi FM, Duhier FMA (2018). Cytochrome P450: Polymorphisms and Roles in Cancer, Diabetes and Atherosclerosis. Asian Pac J Cancer Prev.

[121] Wang LV, Hu SJs (2012). Photoacoustic tomography: in vivo imaging from organelles to organs. Science.

